# Mono-6-Substituted Cyclodextrins—Synthesis and Applications

**DOI:** 10.3390/molecules26165065

**Published:** 2021-08-21

**Authors:** Petr Kasal, Jindřich Jindřich

**Affiliations:** Department of Organic Chemistry, Faculty of Science, Charles University, Hlavova 8, 128 43 Prague 2, Czech Republic; petr.kasal@natur.cuni.cz

**Keywords:** cyclodextrins, mono-6-substitution, synthesis, applications

## Abstract

Cyclodextrins are well known supramolecular hosts used in a wide range of applications. Monosubstitution of native cyclodextrins in the position C-6 of a glucose unit represents the simplest method how to achieve covalent binding of a well-defined host unit into the more complicated systems. These derivatives are relatively easy to prepare; that is why the number of publications describing their preparations exceeds 1400, and the reported synthetic methods are often very similar. Nevertheless, it might be very demanding to decide which of the published methods is the best one for the intended purpose. In the review, we aim to present only the most useful and well-described methods for preparing different types of mono-6-substituted derivatives. We also discuss the common problems encountered during their syntheses and suggest their optimal solutions.

## 1. Introduction

Cyclodextrins (CDs) are supramolecular hosts with a long history [1,2] developed from compounds of just a scientific interest into the compounds manufactured in tons quantities and used [3] in pharmaceutical [4,5], cosmetic [6], or food [7] industries. Native CDs are cyclic oligosaccharides based on glucose (Glc) units manufactured by enzymatic conversion of starch. The most common CDs contain 6, 7, or 8 Glc units and are called α-, β-, and γ-CD, respectively. Their structure reminds a truncated cone with a relatively lipophilic cavity (surrounded by C-O-C and C-H bonds) and hydrophilic edges (formed by OH groups). 

**Figure 1:**
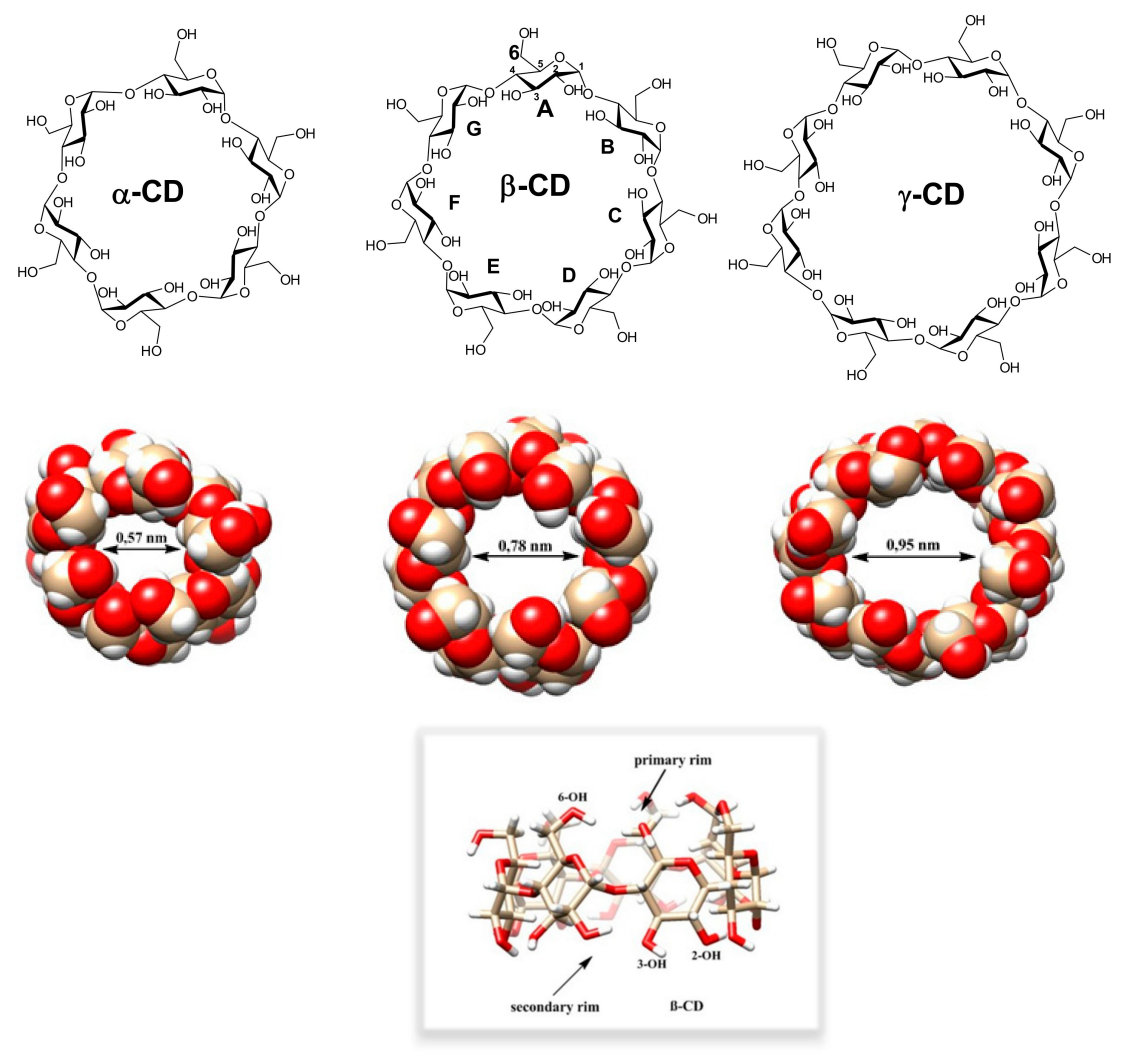
Structure, the numbering of glucose units (A, B, etc.) and atoms (1, 2, etc.), and 3D models of native cyclodextrins.

This unique CD structure (Figure 1) is responsible for its most important property—the inclusion binding of lipophilic guest molecules into the CD cavity [8,9]. By forming the inclusion complex, the properties of guest molecules are modified in various ways. An increase of water solubility [5], modification of spectral properties [10], chemical stabilization [11], and a decrease of volatility [12] can be named as the most utilized phenomena connected with the inclusion complexation.

Nevertheless, the native CDs have limitations, such as solubility, selectivity, or strength of inclusion binding, which are being addressed by preparing various CD derivatives. Due to the large number of OH groups in the CD molecule, the easiest modification method is random derivatization which is the cheapest and used most often for industrial applications. Randomly methylated [13], hydroxypropylated [14], carboxymethylated [15], sulfobutylated [16], or sulfated [17] CDs are the most used derivatives of this type. Products manufactured by these methods are always mixtures of many regioisomers and homologs usually characterized by the average degree of substitution (DS).

More complicated methods are necessary to prepare a so-called “single isomer” CD derivative (i.e., pure chemical compound). The simplest of them is persubstitution based on the reaction of all OH groups of CD with the same reagent. More sophisticated methods then use either regioselective substitution procedures, high-performance separation methods, or both. The overview of such methods was published recently [18,19].

In this review, we concentrate on methods for preparing mono-6-substituted cyclodextrin derivatives, containing just one substituent per the whole CD molecule in position 6 of one Glc unit, which are relatively easy to prepare. That is why the number of publications describing the preparation of such derivatives exceeds 1400, and the used synthetic methods are often very similar. Nevertheless, it might be pretty demanding to decide which of the published methods is the best one for the intended purpose. 

This review aims to present only the most useful and well-described methods for preparing different mono-6-substituted derivatives. We discuss the common problems for each type of derivative and suggest the optimal methods to prepare it. In addition, we maintain a web page https://cyclodextrins.org/synthesis-of-mono-6-substituted-cyclodextrins (accessed on 15 July 2021) containing many references not mentioned here. Most of the published procedures, found by SciFinder, were included in this web page updated periodically by a Python [20] script using pyzotero [21] API module accessing the data in Zotero [22] bibliographic database. Besides the synthetic methods, we also mention the applications in which the prepared CD derivatives were used.

### 1.1. 6^A^-O-Aryl/alkyl-sulfonates of CDs

**Scheme 1:**
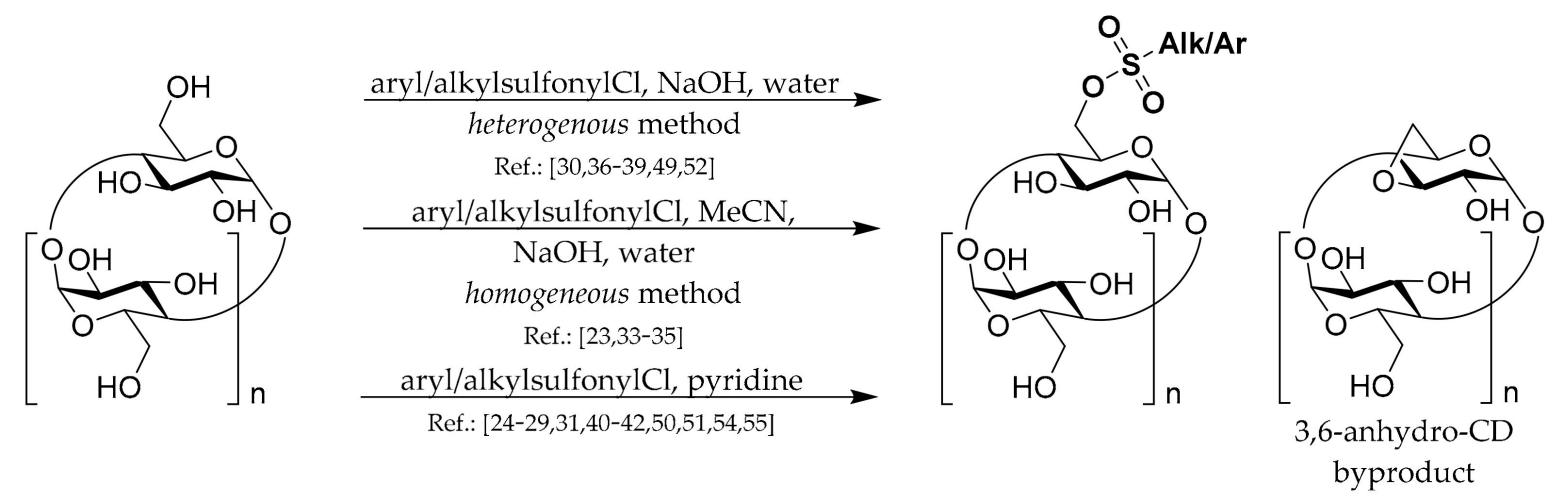
General methods for preparation of 6^A^-*O*-aryl/alkyl-sulfonates of CDs.

To prepare most of the mono-6-substituted CDs, the corresponding mono-arylsulfonate derivatives are used as starting materials. Thus, the review of their preparation methods comes first. General methods for preparation of 6^A^-*O*-aryl/alkyl-sulfonates of CDs are depicted in Scheme 1. The common complication in preparations of all CD monosulfonates is a relatively low yield due to the formation of by-products with more sulfonyl groups and the presence of unreacted CD. Hence, the subsequent purification procedures, repeated crystallization, or chromatography have to be applied if a pure product is needed. Another common danger with the CD sulfonates preparation is the formation of 3,6-anhydro-CD byproducts (Scheme 1), which can lower the yield and be encountered in the presence of strong bases such as NaOH.

The most utilized arylsulfonate α-CD derivative is 6^A^-*O*-(*p*-toluenesulfonyl)-α-CD (Ts-α-CD). There are several articles in which the authors describe its synthesis completely. Synthetic methods can be divided into two major groups according to the used solvent system. One group of authors utilizes NaOH water solution, and the second one performs the reaction in pyridine or its derivatives. The representative of the first group, Xiao et al. [23], who synthesized α-CD-pentacyclic triterpene conjugates as novel potential anti-HCV entry inhibitors, added 1 equivalent of *p*-toluenesulfonyl chloride (TsCl) in acetonitrile into the basic water α-CD solution; the crude product was recrystallized from hot water and obtained in 13% yield. This method has two disadvantages: firstly, the product’s high solubility in an aqueous solution leading to difficulties in the product precipitation, and secondly, Ts-α-CD is readily hydrolyzed in water. However, the vast majority of chemists use the second, *pyridine*, method. 

Melton and Slessor [24] described the synthesis at the beginning of the 70 s. They published the methodology for cyclodextrin mono-6^A^-*O*- modifications, including *p*-toluenesulfonylation (tosylation), azidation, halogenation, and amination. For the tosylation, they used 21 eq. of TsCl in pyridine at room temperature and stopped the reaction after 40 min. According to TLC, the yield of the product was 67%, and final purification was done with an activated charcoal column. The product was obtained in 47% yield. Other authors followed their strategy but with considerable differences in the molar amount of TsCl, purification methods, and yields. 

Brown et al. [25] synthesized amino-derived α- and β-CDs to test their properties and inclusion complexation abilities. They used only 5 eq. of TsCl, a reverse-phase column chromatography (RPC) purification and isolated the product in 20% for Ts-α-CD. Ts-β-CD was purified by recrystallization from water and obtained in a 30% yield.

Tang et al. [26] described a detailed protocol for the synthesis of Ts-CDs, and their subsequent azidation and reduction. They applied 0.9 eq. of TsCl, let the reaction run for one day, and isolated the product in 33% yield, even though they used recrystallization in water to purify the product.

Chwalek et al. [27] prepared click-assembled oligorotaxanes based on lactosyl-α-CD conjugates and biologically evaluated them. They used 10 eq. of the reagent, quenched the reaction after 15 min, and isolated the product in 26% yield after column chromatography utilizing the MeCN/water mixture. 

In the two most recent papers, the authors worked with 5 eq. of TsCl in pyridine or 4-methylpyridine, stopped the reaction after 1 to 3 h, and purified the product using an RPC macroporous adsorption resin, and obtained Ts-α-CD in the yield of 15 and 24% [28,29]. Using 4-methyl-pyridine instead of the unsubstituted one does not have any noticeable impact concerning the yield. 

It is also apparent that there are considerable differences in the number of TsCl equivalents used by different authors. According to our experience, the reason for this is water in pyridine and native α-CD. Due to non-dry conditions, more TsCl needs to be added because it first reacts with the present water, and only after this “drying with the help of the reagent” the rest of TsCl reacts with α-CD. To conclude this paragraph, the optimal and the most detailed procedure for preparing Ts-α-CD is described in the paper published by Tang et al. [26]. 

Fujita et al. [30] prepared three different derivatives, specifically 6^A^-(1-naphthalenesulfonate)-α-CD, 6^A^-(2-naphthalenesulfonate)-α-CD, and 6^A^-(3-nitrobenzenesulfonate)-α-CD. The authors tested conditions for sulfonation reactions and their regioselectivity. All reactions were done in water with NaOH at room temperature. Yields of all three derivatives were around 1%. The authors also tried to prepare 6^A^-(4-nitrobenzenesulfonate)-α-CD but obtained only C-3 and C-2 regioisomers. This work demonstrates another problem with arylsulfonations of α-CD in water (not observed for β-CD)—formation of other regioisomers, in addition to low yields due to easy hydrolysis of the sulfonate.

Only one article describes the complete preparation of 6^A^-(2,4,6-trimethylbenzenesulfonate)-α-CD. Fujita et al. [31] synthesized 6-*O*-polysulfonylated α-CDs derived from mesitylenesulfonyl chloride, separated them, and assigned their regiochemistries and performed the reaction in pyridine with 9 eq. of sulfonyl chloride at room temperature for 1 h. The product was purified using an RPC and obtained in almost 25% yield. 

Synthesis of 6^A^-(methanesulfonate)-α-CD was described in one article, but the derivative was not prepared by direct sulfonylation of native α-CD. Perbenzylation, selective mono-debenzylation, sulfonylation, and total debenzylation strategy were utilized instead [32]. The authors studied cubane type [Fe_4_S_4_(SR)_4_]^2−^ clusters and utilized α-CD dithioester and thiol derivatives stabilization ligands.

The 6^A^-*O*-(*p*-toluenesulfonyl)-β-CD (Ts-β-CD) is the most utilized derivative from this chapter. Unlike its α-CD analog, the synthesis of Ts-β-CD is more diverse in terms of reagents. In the literature, three tosylating agents in connection with β-CD can be found, TsCl, *p*-toluenesulfonic anhydride (Ts_2_O), and (*p*-toluenesulfonyl)imidazole (TsIm). 

Most authors used TsCl as a tosylating agent but applied several different methods. The first method could be named the *homogeneous* method. The general procedure is this: native β-CD is dissolved in an aqueous NaOH solution, TsCl is dissolved in MeCN and added dropwise to the β-CD solution. The formation of turbidity is observed. After an appropriate time (few hours usually), the mixture is filtered, neutralized, or even acidified to induce precipitation of the product, oversulfonated by-products, and unreacted β-CD. The crude is filtered and, in the ideal case, purified by crystallization, column chromatography, or other methods. Unfortunately, most published procedures do not purify the mixture after precipitation. The intended use can justify it, but authors often do not mention impurities consisting of by-products and unreacted starting material. 

Petter et al. [33] were the first who fully described this procedure in their paper describing aggregation and inclusion complexation of alkylamino-β-CDs. The authors utilized 30 eq. of NaOH and 1 eq. of TsCl, stopped the reaction after 2 h of stirring at room temperature and induced the crude’s precipitation by lowering the temperature to 0 °C instead of lowering the pH. This is probably why the authors isolated only 11% of the impure product. Vizitiu et al. [34] have optimized Petter’s procedure, primarily by including acidifying steps and recrystallization of the crude from hot water. 

Jicsinszky and Iványi [35] formed the complex of β-CD with toluene before the reaction. The authors purified the crude by repeated crystallization from 50% aq. EtOH and isolated the product in a 34% yield.

The second method could bear the name *heterogenous*. The difference is the addition of TsCl in a solid form to a water solution of β-CD; after stirring, aqueous NaOH solution is added. The mixture is filtered after 10 min usually, and the rest is the same as described for the homogeneous method. In our experience, it is better to crush the reagent in mortar. Otherwise, the reaction is not reproducible. 

Hacket et al. [36] studied the complexation of di-and tripeptides by alkylamino-β-CDs. They fully described the CD tosylation using 13 eq. of NaOH and 6 eq. of TsCl. The reaction was performed at 0 °C; the crude was recrystallized from hot water and isolated in a 22% yield. The authors stated that the purity of the product was 92% according to NMR, and further crystallizations did not have any effect. McNaughton et al. [37] utilized the same strategy but used ammonium chloride for neutralization. 

Tripodo et al. [38] used a strong cation exchanger in H^+^ form for neutralization of the aq. NaOH solution. The advantage could be easier removal of the salt from the reaction mixture by simple filtration of the exchanger. 

At last, we should mention the article published Novokshonov et al. [39]. From the literature, chemists can deduce that yields of Ts-β-CD preparation range from 10 to 30% mostly, these authors stated yield 58%. This surprisingly high yield is the result of reusing the unreacted native β-CD and TsCl one more time. Both product fractions were then combined, and this is the reason for such a high yield. 

The third method is based on utilizing pyridine as a solvent and a base. This method is mainly used for α- and γ-CD derivatives because due to their higher water solubility, precipitation of the crude from water solution is insufficient. When the tosylation in pyridine is completed, pyridine is distilled off, and crude is precipitated in acetone. Again, as mentioned above, for the homogeneous method, precipitation is not satisfactory to get a pure product, and another purification method should be utilized. 

Defaye et al. [40], in their publication about glucopyranosyl-β-CD conjugates and their inclusion complexation studies, were among the first to describe this procedure for β-CD. Pyridine solution of TsCl was added to the pyridine solution of β-CD at 0 °C. The reaction ran overnight; after pyridine evaporation, the crude mixture was precipitated in ether. The product was purified by repeated recrystallization from hot water and isolated in a 26% yield. 

In the same year as Defaye, Ekberg et al. [41] published their article describing a similar protocol. Their work dealt with tripeptide-β-CD conjugate synthesis and its artificial enzyme utilization in ester hydrolysis and amide bond formation reactions. The main difference was the purification strategy. Ekberg purified the product by preparative TLC and isolated the product in a 3.4% yield. Sforza et al. [42] purified the crude mixture by preparative HPLC and obtained a 29% yield. They also isolated three regioisomers of 6-*O*-disubstituted homologs and studied them by fragmentation and ESI-mass spectrometry. 

Some authors worked with TsIm instead of TsCl. Byun et al. Reference [43] performed the reaction in water by the identical strategy already described in the paragraph about the heterogeneous method. The authors isolated the crude mixture by precipitation in acetone and obtained the impure product in a 40% yield. Trotta et al. [44] performed the reaction in a cavitating-tube reactor applying sonification. The crude mixture was purified only by precipitation in acetone again, and the yield of the impure product was 55%.

Zhong et al. [45] published an article in which Ts_2_O was used instead of TsCl. Again, the procedure was identical to the one described above for the heterogeneous method. The authors stated they isolated the pure product in a 61% yield after precipitation in acetone. In this case, some authors later reported the non-reproducibility of this protocol [36].

Another group of authors utilized *more doses* of TsCl to improve yields. Brady et al. [46] dissolved β-CD in an aqueous NaOH solution, cooled the solution to 0 °C, and added the first dose of TsCl (2.4 eq.) After 2 h, the second dose of TsCl (3.6 eq.) was added, and the reaction was quenched after 3 h. The product was purified by repeated crystallization from hot water and isolated in a 25% yield. 

Xu et al. [47], in their paper dealing with a CD-based sensor for ferric ion detection, utilized the same procedure but added even three doses of TsCl (1.8, 1.8, and 2.4 eq.) and isolated the product in a 31% yield after repeated hot water crystallizations. From these results, it is evident that there is no significant increase in the yield comparing this strategy to the heterogeneous method.

The methods of the last group do not fit into any of described categories. Law et al. [48] performed the tosylation in the presence of Cu(II) salts in an aqueous NaOH solution. The yield and regioselectivity were improved by forming chelate between copper ion and secondary hydroxyls. The yield was 35% after repeated crystallizations from 50% aq. *n*-propanol. 

To mention other 6^A^-*O*-(aryl/alkylsulfonate)-β-CDs, 6^A^-*O*-(4-carboxybenzenesulfonate)-β-CD was synthesized by Wang et al. [49]. They described the preparation of a photoresponsive molecularly imprinted system based on host–guest interactions between grafted azobenzene derivative and β-CD derivative on silicon. The CD sulfonylation was done in water with NaOH and 1 eq. of sulfonyl chloride for 2 h at 30 °C. The yield was 8% after precipitation. 

Another two derivatives, 6^A^-*O*-(9,10-dihydro-9,10-dioxo-1-anthracenesulfonate)-β-CD and 6^A^-*O*-(9,10-dihydro-9,10-dioxo-2-anthracenesulfonate)-β-CD, were prepared by Aquino et al. [50]. They used them as intermediates for anthraquinoine-modified CDs and their photochemistry studies. Both reactions were done in pyridine with 1 eq. of sulfonyl chloride. Reactions were performed at 0 °C and ran for 2 h. Yields were estimated at around 20% after preparative HPLC. 

Sforza et al. [42] synthesized 6^A^-(2,4,6-trimethylbenzenesulfonate)-β-CD. The authors did the reaction in pyridine with 2 eq. of sulfonyl chloride at RT for 24 h. Preparative HPLC purification afforded product in 19% yield.

Concerning the 6^A^-*O*-(*p*-toluenesulfonyl)-γ-CD (Ts-γ-CD), Fujita et al. [51], in their publication focusing on enzymatic hydrolysis of arenesulfonyl-γ-CD, synthesized this compound in 1988 utilizing the pyridine method described in the previous chapter about β-CD analogs. That means he used pyridine and 8 eq. of TsCl and isolated the compound in a 31% yield applying RPC. 

The heterogeneous method, including NaOH water solution and solid TsCl, typical for β-CD analog, was utilized by Van Guyse et al. [52] in their publication concerning the preparation of fullerene-γ-CD nanoparticles with potential biomedical applications. After precipitation from acetone, the authors obtained the product in a 54% yield. However, the authors acknowledged the presence of native γ-CD, and the actual yield was 28% based on NMR. 

Pham et al. [53] developed a completely different strategy during the synthesis of diamide-linked γ-CD dimers. The reaction was performed in DMF, and dibutyltin oxide, together with triethylamine, was included. TsCl was added in two portions, and after crystallization and column chromatography separation, 6^A^-*O*- and 2^A^-*OTs*-γ-CD were obtained in a 4 and 8% yield, respectively.

About other compounds which fit into this chapter, Palin et al. [54] prepared a series of these derivatives to investigate how the type of sulfonylation agent can influence the yield of a mono-substituted γ-CD derivative. Authors prepared 6^A^-*O*-(4-carboxybenzenesulfonate)-γ-CD, 6^A^-*O*-dansyl-γ-CD, 6^A^-*O*-(2-naphthalenesulfonate)-γ-CD, 6^A^-*O*-(2,4,6-trimethylbenzenesulfonate)-γ-CD, and 6^A^-*O*-(2,4,6-triisopropylbenzenesulfonate)-γ-CD (Trips-γ-CD). The last one was obtained in a 69% yield. According to the authors, 2,4,6-trisisopropylbenzenesulfonyl chloride (TripsCl) is the best sulfonating agent for the preparation of 6^A^-*O*-arylsulfonate-γ-CDs in terms of monosubstitution. However, this yield was obtained after simple precipitation. Due to that, we can expect the product was not of sufficient purity. The authors obtained only 9% of a sufficiently pure product after crystallization of the crude product.

Derivatized 6^A^-*O*-biphenylsulfonate-γ-CD was prepared by Yang et al. [55]. Their paper studied the influence of the inclusion complexation of 2-anthracenecarboxylic acid with γ-CD derivatives on the photocyclodimerization. They used the standard CD sulfonylation method with pyridine and an appropriate sulfonating agent. The product was purified by RPC and obtained in a 24% yield. 

In summary of this chapter, we include our notes along with peculiarities or even common mistakes; this could be useful for anyone who will synthesize and work with these types of CDs derivatives.

In all mentioned methods, authors use from 0.5 eq. to even 9 eq. of tosylating agent, but there is no direct correlation between the molar amount and the product’s yield. In our experience, 1 eq. of tosylating agent is a sufficient amount for *heterogeneous* method if the solid reagent is sufficiently crushed. 

The method for purification of the product is much more important than the amount of tosylating agent. The common fact is that many authors purify the product just by simple precipitation from acetone or directly from the reaction mixture after concentrating the solution. In our experience, the product still contains after precipitation a large amount of starting β-CD and over-tosylated by-products. Some authors even mention this fact in their papers [36,42,48]. The simplest way of purification is recrystallization from water or aqueous solutions [34,35,36,48]. Some authors also use chromatography separation [42], and in one article, authors even used preparative TLC [41].

Of course, yields differ according to the used purification procedure. If only precipitation is used, yields can be higher than 50% [44,45], but this is never a pure product. On the other hand, if a proper purification method is used, yields are usually in the range of 20-35% [46,47]. In our experience, the most practical purification method of Ts-β-CD consists of the three times repeated crystallization of the crude product from 50% MeOH/water solution giving an overall yield of around 25% and removing reliably unreacted β-CD and over-tosylated by-products [56].

In the end, Table 1 summarizes all described synthetic methods, the number of articles, yields, and purification techniques.

### 1.2. 6^A^-Deoxy-6^A^-halogens of CDs

**Scheme 2:**
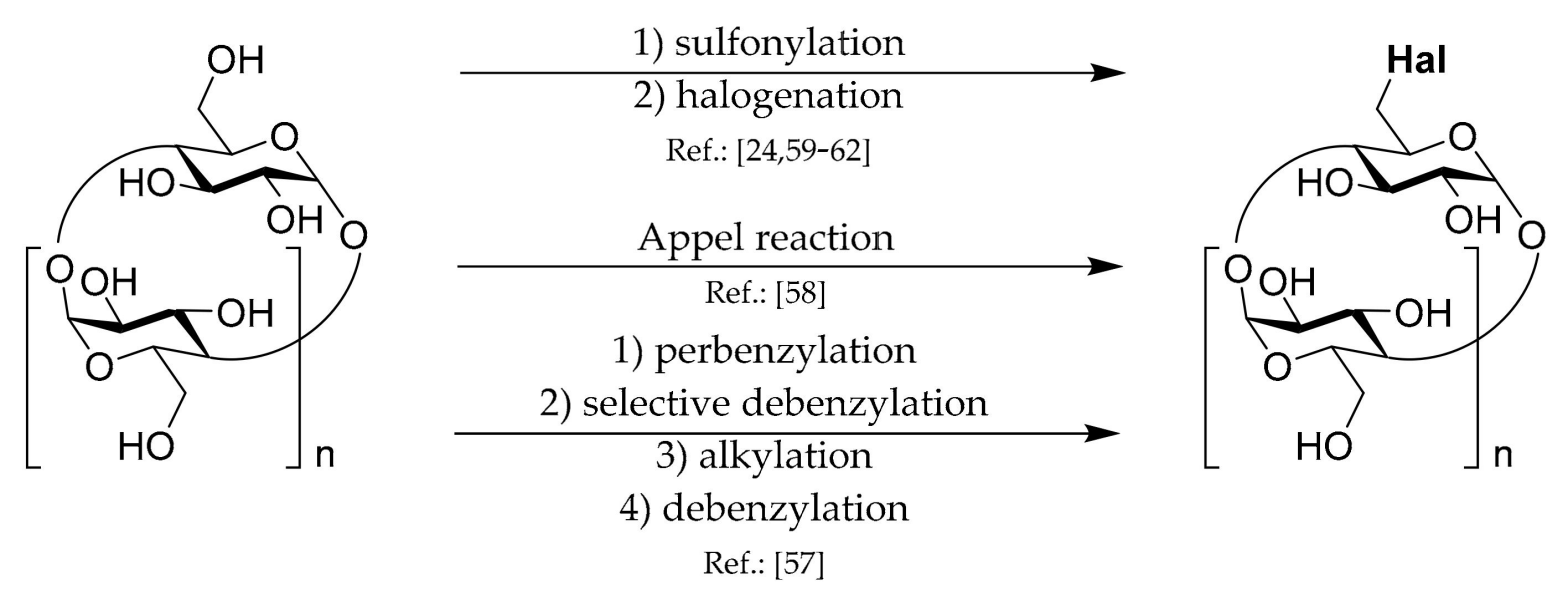
General methods for the preparation of 6^A^-deoxy-6^A^-halogens of CDs.

General methods for the preparation of this type of CD derivatives are shown in Scheme 2.

Concerning the α-CD derivatives, Melton and Slessor [24] prepared the chloro, bromo, and iodo derivatives. Chloro derivative was prepared by heating (100 °C) Ts-α-CD in DMF with 10 eq. of tetrabutylammonium chloride for 1.5 h. The final product was purified by pouring onto a strong cation exchanger followed by a strong anion exchanger and obtained in a 56% yield. Bromo derivative was synthesized by a very similar procedure with 50 eq. of LiBr and obtained in an 89% yield. Iodo derivative was prepared by refluxing Ts-α-CD with 30 eq. of NaI in water for 1 h; the product was purified by crystallization from water and obtained in a 52% yield. 

Fredy et al. [57] developed polyrotaxanes based on BODIPY-modified CDs and their Gd^3+^ complexes as potential imaging agents. They prepared the CD chloro derivative utilizing perbenzylation/selective debenzylation strategy resulting in a perbenzylated α-CD with one free hydroxyl group at the primary rim. This compound was further methanesulfonylated, and subsequently, the methanesulfonyl group was substituted by chlorine by heating with Et_3_N.HCl. The product was purified by flash column chromatography. In the end, the remaining benzyl groups were removed by hydrogenation. 

Kumprecht et al. [58] synthetized bromo analog by Appel reaction utilizing CBr_4_ and Ph_3_P (both 5 eq.) as an intermediate in disulfide bonds-based α-CD duplex, in which inclusion complexation abilities were studied. The product was purified by RPC and obtained in a 31% yield.

More articles are about β-CD halogeno derivatives. Several authors published a synthesis of the iodo derivative. Ye et al. [59] utilized microwave irradiation instead of classical oil bath heating to synthesize this compound. The authors aimed to modify β-CD with *R*-(−)-2-phenylglycinol and test its chiral discrimination capabilities upon amino acid complex formation. Ts-β-CD and KI (10 eq.) were dissolved in DMF, irradiated for 6 min (475 W). The final product was purified by recrystallization from a butanol-alcohol-water mixture and obtained in an 85% yield. 

Ogoshi et al. [60] have synthesized poly(phenylene ethynylene) carrying β-CD. Then they evaluated it as a fluorescent chemosensor. For I-β-CD preparation, they utilized classical oil bath heating, dissolved Ts-β-CD and KI (16 eq.) in DMF, and stirred the mixture for 6 h at 100 °C. The final product was precipitated by tetrachloroethylene from a water solution and isolated in an 82% yield.

Rodríguez-Lavado et al. [61] used a similar strategy in their work about mannosylated CDs and their application for targeted delivery. They used NaI (5 eq.), stirring overnight at 80 °C, precipitation with the tBuOH/EtOH/water mixture, and obtained the product in a 90% yield.

Shipilov et al. [62] prepared both bromo and iodo derivatives as intermediates for monocationic CD derivatives. Melton and Slessor’s method [24] inspired the authors. However, they applied significant modifications. In the case of bromo derivative, authors used KBr instead of LiBr and only 2 eq. instead of 50 eq. The product was purified by precipitation from a small amount of water and isolated in an 85% yield. Iodo derivative was prepared in a more similar way to Melton and Slessor’s method compared to bromo derivative. However, instead of crystallization, the product was precipitated from acetone and obtained in a 79% yield.

No articles concerning the γ-CD derivatives have been found to this day.

Table 2 summarizes all described synthetic methods; the number of articles, yields, and purification techniques is included.

### 1.3. 6^A^-O-Aryls/alkyls of CDs

**Scheme 3:**
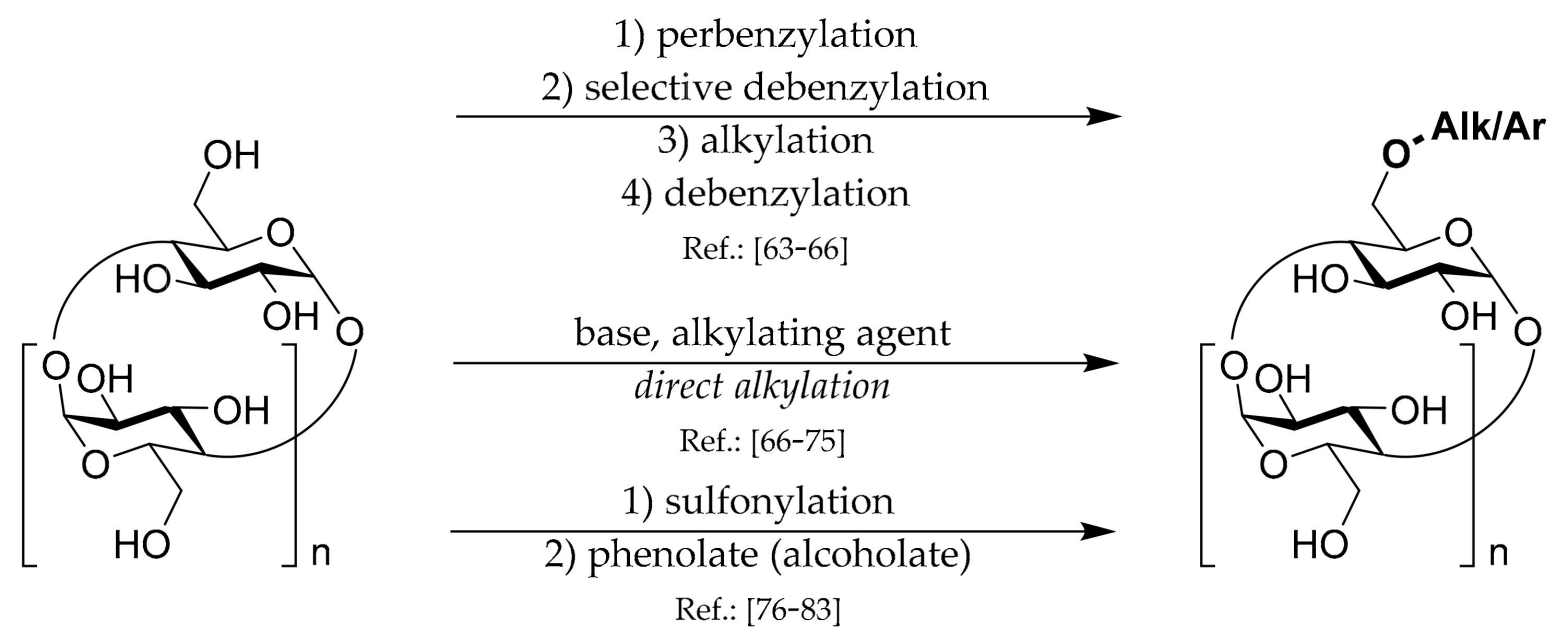
General methods for preparation of 6^A^-*O*-aryls/alkyls of CDs.

Synthetic strategies leading to these types of CD derivatives can be divided into several groups which are depicted in Scheme 3.

The first, *(de)benzylation,* strategy consists of perbenzylation, selective debenzylation, alkylation, and debenzylation steps. Yields for each step are high or even quantitative. Rousseau et al. [63] utilized a bis-debenzylated-α-CD derivative to prepare acetone-bridged CD derivatives suitable as catalysts for epoxidation reactions. The reaction was performed in DMF with NaH and alkyl chloride at room temperature overnight. The product was purified by column chromatography and obtained in an 84% yield. 

Hauch Fenger et al. [64] used a mono-debenzylated-α-CD derivative in THF. The authors added tBuOK and Bu_3_NI at 0 °C and alkyl bromide in the end. The mixture was stirred at room temperature overnight. The crude was purified by flash column chromatography and isolated in a 77% yield. In the end, the authors synthesized CD derivatives bearing carbonyl groups and mimicking oxidases.

Yamanoi et al. [65] used a mono-debenzylated-β-CD derivative in DMF with 120 eq. of KOH as a base and 4.5 eq. of alkyl iodide. The reaction was stirred at room temperature for 24 h. The final product was purified by preparative silica gel TLC and obtained in a 51% yield. The authors aimed to prepare a glucose-modified β-CD, test its complexation with anticancer agent doxorubicin, and its ability to work as glycosyl acceptors for the transglycosylation reactions.

Zhou et al. [66] described tin-containing CDs with the potential to function as a radical halides reductant. They also utilized mono-debenzylated-β-CD derivative in DMF with only 5 eq. of NaH as a base and 1 eq. of alkyl bromide. After 2 h at room temperature, the final product was purified by column chromatography and isolated in an 82% yield.

The second strategy is the *direct alkylation* of native α-CD. The advantage is only one reaction step compared with four reaction steps in the first described strategy. Disadvantages are lower yields, around 15–20%, due to the formation of regioisomers and overalkylation side-products. Especially the question of regioisomers can be an issue. There are several strategies under which one regioisomer should be the major one, but none can be considered a general method. Chemists cannot avoid purification if they need pure regioisomer of mono-alkylated α-CD. These purification techniques can include acetylation of the remaining hydroxyl groups, regioisomers column separation, and deacetylation/deprotection. 

We described that strategy [67] for mono-6-allylation. We conducted the reaction in 8M NaOH, which also ensured deprotonation of less acidic 6-OH groups resulting in pure 6^A^-*O*-alkylation (no 2^A^-*O* or 3^A^-*O*) for this particular set of compounds and reagents. The yield of the purified product was 14%; the allyl group was used for the attachment of fluorinated chains to the 6^A^-*O* position using cross-metathesis. 

Liu et al. [68] used the same strategy with different alkyl chloride (coumarin chloride), and after purification by precipitation, the authors obtained the product in a 50% yield. The authors tested its potential as a dispersant for CNTs by adsorption onto the surface of CNTs. 

Tian et al. [69], in their paper concerning synthetic strategies for selective CDs modifications, utilized a slightly different approach. Native α-CD was dissolved in 2,6-lutidine, and substituted benzyl chloride was added. The mixture was heated to 150 °C and stirred for 3.5 h. Lutidine reacted with the benzyl chloride, and the formed lutidinium salt reacted with CD hydroxyl groups. The final product was purified on the Sephadex column and isolated in a 34% yield.

Several authors used the direct alkylation strategy for native β-CD. Lang et al. [70] performed a nucleophilic aromatic substitution reaction. Deprotonated β-CD reacted with porphyrin derivative possessing pentafluorobenzene rings in DMF at room temperature. The final product was purified by RPC and obtained in a 14% yield. Photoinduced electron transfer between this porphyrin-CD conjugate and various guest molecules was investigated by fluorescence spectroscopy.

Barata et al. [71] conducted the same type of reaction with corrole derivative and slightly modified conditions. They synthesized corrole-CD conjugate and then tested its in vitro efficiency towards tumoral HeLa cells. DMSO was used as a solvent instead of DMF, and potassium carbonate was utilized instead of sodium hydride. The final product was purified by RPC and isolated in a 23% yield. 

We also prepared 6^A^-*O*-allyl-β-CD by the already mentioned strategy utilizing an excess of NaOH [72]. Under these conditions, mono 6^A^-*O*- regioisomer is the main product. Nevertheless, the peracetylation, chromatographic separation, and deacetylation steps were needed to isolate pure 6^A^-*O*-regioisomer in a 6% yield. 

Zhou et al. [66] utilized practically the same approach but with propargyl bromide. Purification via peracetylation was included, and the peracetylated 6^A^-*O*-regioisomer was obtained in a 13% yield. 

Liu et al. [73], during their work on crown ether-modified CDs synthesis and molecular binding behavior with fluorescence organic dyes, performed a similar type of synthesis with benzyl bromide derivative in DMSO with grounded NaOH. The reaction mixture was stirred at 55 °C for 4 h; the product was purified by Sephadex column chromatography and isolated in a 20% yield. However, without the peracetylation strategy mentioned above, the product is with high probability contaminated with different regioisomers. 

Novokshonov et al. [74] studied the regioselectivity for the reaction of native β-CD with allyl bromide and concluded that it depends heavily on the solvent. In DMSO, the mono-substituted product contained 2^A^-*O*-regioisomer almost quantitatively, but in DMF, more than 95% of 6^A^-*O*-regioisomer was formed.

Using the direct alkylation strategy and using similar conditions as for α-CD, we prepared 6^A^-*O*-allyl-γ-CD [67] and 6^A^-*O*-propargyl-γ-CD [75] in 18% and 13% yield, respectively.

The third strategy for preparation of 6^A^-*O*-alkyl-CDs, *Ts-CDylation*, i.e., the reaction of 6^A^-*O*-Ts-CDs with the corresponding alcoholate, cannot be used for aliphatic alcohols. The alcoholate is not a good enough nucleophile (compared to amines and thiols) and reacts as a base preferentially. The high basicity leads to the deprotonation of CD hydroxyl groups, intramolecular nucleophilic substitution, and the formation of 3,6-anhydro-α-CD as the main product. Aromatic alcohols (phenols) are the exception due to their lower basicity and enhanced nucleophilicity if they have proper substituents. 

Zhang et al. [76] described reactions of compounds containing a phenolic hydroxyl group giving yields higher than 50%. The authors synthesized manganese porphyrin-α-CD and studied its potential to catalyze epoxidation reactions.

Several authors published the use of Ts-CDylation strategy for β-CD. They utilized Ts-β-CD as starting electrophilic compound and proper phenolate as a nucleophile. Liu et al. [77] performed the reaction of Ts-β-CD with azobenzene derivative possessing phenolic hydroxyl group in the presence of K_2_CO_3_. The product was isolated by precipitation from acetone and obtained in a 78% yield. Its complexation behavior with aliphatic alcohols was studied.

Casas-Solvas et al. [78] used the same azobenzene derivative possessing phenolic hydroxyl group derivative as Liu and performed the same reaction under the same conditions. After proper purification, including precipitation, Soxhlet extraction, and column chromatography separation, the authors obtained only 39% of the product and 55% of the 3,6-anhydro-β-CD by-product. These results indicated that Liu’s product was not pure. The authors also tested cesium carbonate as a base using the same purification techniques. They obtained the product in a 61% yield with a 38% yield of the 3,6-anhydro-β-CD by-product. 

Liu et al. [79] also prepared series of 6^A^-*O*-aryl-β-CDs from various phenol derivatives to study their inclusion complexation behavior in solution and self-assembling behavior in the solid-state. The general protocol could be described as follows: phenol derivative (2 eq.) and K_2_CO_3_ (2 eq.) were dissolved in DMF and stirred at room temperature for 2 h. The solution of Ts-β-CD in DMF was added, and the mixture was stirred for 24 h at 80 °C. The final products were purified by the Sephadex column, and the yield varied from 15 to 36%. 

Puglisi et al. [80] worked with the porphyrin derivative bearing phenol group. This compound was added to the DMF solution of Ts-β-CD containing Cs_2_CO_3_. The reaction mixture was heated to 140 °C and stirred for 1 day. The product was purified by preparative HPLC and isolated in a 30% yield. Its self-association behavior without or in the guest’s presence was investigated.

Fraix et al. [81] also utilized a porphyrin derivative with a phenol group, like Puglisi et al., for synthesis of porphyrin-CD conjugate, complexation with nitric oxide photo donor, and utilization of this aggregate as nitric oxide and singlet oxygen generator in cells. However, the synthesis and purification method strategies are significantly different. The reaction was done in DMSO at room temperature for 4 days and the product purified by a combination of dialysis and column chromatography and obtained in a 37% yield. 

Zhao et al. [82], on the other hand, worked with aliphatic alcohols, specifically ethylene, diethylene, and triethylene glycol, purified the products by column chromatography, and obtained them only in 10, 12, and 14% yield, respectively. The authors tested inclusion complexation abilities of these dimers with various organic dyes.

Only one article describes the utilization of the Ts-CDylation strategy based on 6^A^-*O*-aryl/alkylsulfonate-γ-CD. Park et al. [83] reacted 6^A^-*O*-(2-naphthalenesulfonate)-γ-CD with 6-hydroxy-2-naphthalene sulfonate, a compound possessing phenolic hydroxyl group, and obtained the product in 24% yield after the purification by anion-exchange column chromatography and ultrafiltration. Together with another prepared pyrene-derived amido-γ-CD, the authors studied their homodimerization and heteroassociation behavior.

The last strategy, *photolysis*, was described just in one publication, and the CD derivative was prepared via solid-state synthesis. Krois et al. [84] formed a complex between β-CD and an azaadamantane derivative. After photolysis, the product was purified by preparative HPLC and obtained in 10% yield.

To sum up the alkylation/arylation methods, (de)benzylation strategy gives the highest yields, despite consisting of four steps. This disadvantage is overcome by the fact that only 6^A^-*O*- regioisomer can be formed. Therefore, no time-consuming regioisomer separations are necessary. Most authors used the direct alkylation strategy, although in most cases, a mixture of regioisomers of monosubstituted products is formed. If a pure compound (in terms of regioisomers) is not needed, just the purification from over-alkylated side-products and native CD is necessary. If a pure regioisomer is needed, then the regioisomers’ mixture usually has to be separated via peracetylation procedure. Only a few authors use Ts-CDylation of aliphatic alcoholates due to the formation of 3^A^,6^A^-anhydro-CD is the main product. Compounds with phenolic hydroxyl groups are an exception and can be used together with 6^A^-*O*-(arylsulfonate)-CDs. Table 3 gives an overview of the used strategies.

### 1.4. 6^A^-O-Acyls of CDs

**Scheme 4:**
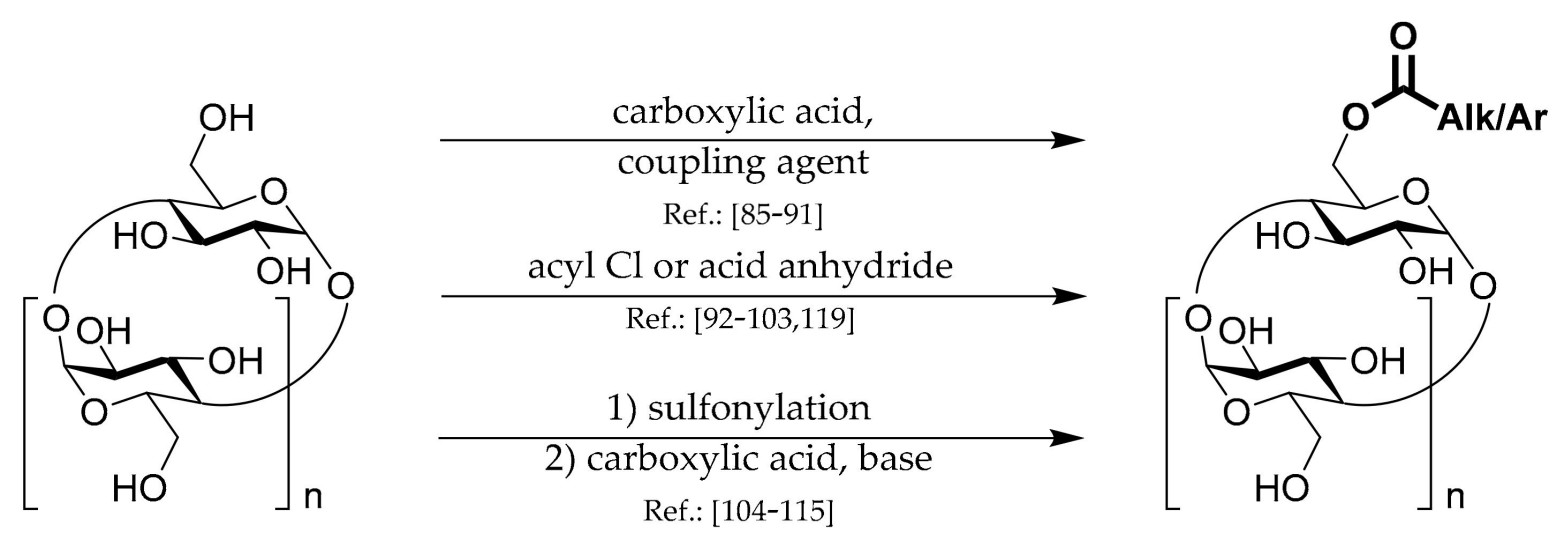
General methods for preparation of 6^A^-*O*-acyls of CDs.

Three general strategies for the preparation of this type of CD derivatives are shown in Scheme 4.

The first strategy uses standard *coupling* chemistry based on carbodiimide, DMAP, CD, and appropriate carboxylic acid. Liang et al. [85] performed the reaction with α-, β-, or γ-CD, tetraphenylethene carboxylic acid derivative, and DCC. The reaction mixture was stirred for 3 days at room temperature. The product was precipitated from diethyl ether and obtained in 52% to 70% yield, depending on the starting CD. The product was utilized as a single molecular luminogen with a cell imaging potential due to an intramolecular host–guest interaction between one of the phenyl rings and CD cavity, which restricted the motion, resulting in enhanced fluorescence.

Deng et al. [86] connected sulfobetaine and α-CD via ester bond by using EDC and DMAP. Again, the final product was isolated by precipitation from acetone in a 75% yield. Later, this compound was coupled with doxorubicine and tested for tumor imaging and drug delivery. In both papers, the authors purified products just by simple precipitation, so purity is questionable.

Liu et al. [87] mixed diselenobisbenzoic acid and β-CD in DMF/pyridine mixture. DCC and 4 Å molecular sieves were added, and the mixture was stirred for 12 h at 0 °C and another 18 h at room temperature. Then the mixture was let stay for 3 days until no more precipitate was formed. After filtration of DCU, the crude product was purified by a Sephadex column and obtained in a 19% yield. The inclusion complexation behavior of this compound with organic dyes was further investigated.

Wu et al. [88] let react spiropyran and β-CD in DMF with an excess of DCC and DMAP. The pure product was obtained by column chromatography purification in a 28% yield. Later, the authors prepared a complex of a rhodamine dye and this photosensitive spiropyran-β-CD derivative. According to the results, a rhodamine fluorescence emission could be switched on and off by vis and UV light and energy transfer between rhodamine and spiropyrane moiety.

Guo et al. [89] also used DCC and DMAP in DMF to bind 2-pyrene butyric acid and β-CD. The product was isolated from the crude by precipitation from acetone and isolated in a 15% yield. The product was utilized by the authors together with azobenzene-modified polymer for nanotube formation based on host–guest interactions.

Michel et al. [90] utilized HOBt, DCC, and DMAP combination in the reaction of β-CD with cationic surfactant propanoic acid derivative. The reaction was performed at 50 °C, and the product was isolated by precipitation from acetone in a 65% yield. The product was further tested as a host molecule for low-solubility drugs utilized for melanoma treatment.

Wang et al. [91] reacted 2,6-naphthalenedicarboxylic acid with γ-CD with the utilization of DCC and HOBt reagents. After 2 days of stirring at room temperature, the formed monoester product was isolated by RPC in a 13% yield. The complexation with anthracenecarboxylate was studied together with its photocyclodimerization leading to enantioenriched head-to-head dimers.

The second, *acyl chloride*, strategy represents the most utilized methodology. Native CD is mixed with acyl chloride (or an active ester) in the presence of pyridine or TEA. Several authors utilized this strategy. Yang et al. [92] added anthracene-2-carbonyl chloride into the pyridine solution of α-CD. The reaction mixture was stirred at room temperature for 2 h. The final product was purified by RPC and isolated in a 5% yield. The compound was used as a supramolecular chiral photosensitizing host for enantiodifferentiating photoisomerization of (*Z,Z*)-1,3-cyclooctadiene to its (*E*,*Z*)-isomer. 

Nakamura et al. [93] utilized a similar approach to connect *p*-chlorocarbonyl phenylboronic acid to α- and β-CD. The authors purified both mixtures by a highly porous polystyrene gel column and obtained products in yields around 11%. The phenylboronic acid-CD conjugates intermolecular interactions and resulting structures were studied. The authors found out that formed polymers or dimers could be disintegrated in the presence of sugars due to boronate–sugar interactions.

Karpkird and Wanichweacharungruang [94] used a derivatized cinnamoyl chloride to synthesize a new α-, β-, and γ-CD derivatives. Then they tested their photostability caused by inclusion complexation. Each reaction was performed in DMF/pyridine 3/1 mixture with DMAP. Products were isolated by precipitation in 15 to 30% yields. 

Edunov et al. [95] have utilized TEA as a nucleophilic catalyst and a base instead of pyridine or DMAP in their work about selectively substituted CDs. The authors performed benzoylation of α-CD in DMF. The final product was also isolated just by precipitation and obtained in an 85% yield. In the last two papers, the authors precipitated the products from the crude mixture, so the purity is questionable. 

Gao et al. [96] synthesized α- and β-naphthoyl-β- and γ-CDs by the reaction of appropriate chlorides with CD in pyridine. Products were purified via precipitation from acetone/water 5/1 mixture or by Sephadex column chromatography and obtained in 15 to 30% yields. 

Liu et al. [97] mixed 2,2’-bipyridine-4,4’-dicarboxylic dichloride and β-CD in DMF/pyridine mixture. The authors added DCC and let the reaction run for 18 h at 0 °C and another 48 h at room temperature. The mixture was allowed to stay until no more precipitate was formed. After filtration of DCU, the crude product was purified by a Sephadex column and obtained in a 30% yield. The complexation behavior of this dimer with organic dyes was further studied and characterized.

Wang et al. [98] performed the reaction between β-CD and *p*-nitrobenzoyl chloride in pyridine. The reaction was done at 0 °C and stirred for 36 h. The product was purified by crystallization from acetone/water 5/1 mixture and obtained in an 18% yield. The authors studied photoinduced electron transfer of this β-CD conjugate. A phenomenon observed due to supramolecular complex formation with naphthalene derivatives.

Chan et al. [99] utilized pyruvic acid chloride (15 eq.) to react with β-CD in DMF/pyridine mixture and DMAP as a nucleophilic catalyst. According to the authors, repeated precipitation of the product from EtOH afforded the product in pure form and a 44% yield. The product was further utilized as an alkene epoxidation mediator in the presence of Oxone.

Kurochkina et al. [100] have prepared acetylsalicyloyl β-CD from the appropriate chloride. The reaction was done in pyridine, and acyl chloride was added in benzene. The product was precipitated from the reaction mixture by pouring into EtOH and isolated in an 86% yield. 

Tang and Li [101] reacted β-CD with maleic anhydride in DMF at 80 °C for 10 h. The product was purified by precipitation from chloroform and obtained in a 62% yield. This product was further copolymerized with *N*-acryloyl-3-aminophenylboronic acid, and its self-assembling behavior was investigated. The drug-release behavior of formed microspheres was also studied.

Ma et al. [102] synthesized Boc-aminobenzoyl-β-CD by the reaction of β-CD with Boc-aminobenzoyl chloride in pyridine at 0 °C and room temperature. The pure product was obtained by RPC and isolated in a 36% yield. After Boc deprotection, PABA-β-CD conjugate was tested as the intestinal delivery system for PABA due to ester linkage hydrolysis in the intestines.

Ueno et al. [103] utilized 1-pyrenylbutanoyl chloride for the reaction with γ-CD. After 2 h of stirring in pyridine at 0 °C, the product was purified by the Sephadex column chromatography. The product was further recrystallized from the ethylene glycol–water mixture and obtained in a 7% yield. The authors investigated its self-association and inclusion complex formation.

The third main strategy is Ts-CDylation of a salt of a carboxylic acid with Ts-CD. Due to the low nucleophilicity of the salts, elevated temperatures are usually necessary. The great advantage is the certainty of obtaining only one regioisomer and no multiply substituted by-products. Hoshino et al. [104] dissolved Ts-α-CD in DMSO, added 4-aminocinnamoyl sodium salt, and let it stir for 24 h at 80 °C. The final product was purified via a semi-preparative HPLC column and isolated in a 19% yield. Then the compound was further treated with 2,4,6-trinitrobenzenesulfonic acid sodium salt to install the stopper. The resulting product could be obtained as monomer or cyclic trimer of [2]rotaxane based on the applied conditions.

Yang et al. [105] utilized a similar strategy with Ts-α-, β-, and γ-CD and 2-naphthyloxyacetate sodium salt in DMSO. After stirring for 48 h at 85 °C, products were purified via RPC and obtained in yields around 7%. The photocyclodimerization of the anthracene-CDs was further tested in the presence of γ-CD or cucurbit [8] uril, giving possible products in a complementary fashion.

El-Kamel et al. [106] synthesized 6^A^-*O*-acyl-α-and β-CDs from two sodium salts (from naproxen or flurbiprofen substituted propionic acid) and Ts-β-CD. The reactions were done in DMF and stirred for 48 h at 100 °C. Products were purified via ion-exchange column chromatography and obtained in yields of 30% and 70%. Products were further tested as potential prodrugs and colon-targeted delivery systems.

Miyauchi et al. [107] used 4-aminocinnamic acid sodium salt for the reaction. The reaction was performed in DMSO at 80 °C for 12 h, and the product was precipitated with acetone in a 55% yield. A trinitrophenyl group was further installed, and the authors observed and studied the formation of supramolecular [2]rotaxane polymer. It was caused by β-CD binding a trinitrophenyl group from another molecule.

Sakuraba and Maekawa [108] performed two reactions with two regioisomeric potassium salts of dibenzyloxybenzoic acids. Reactions were done at 90 °C for 40 h in DMSO; the products were purified by recrystallization from water/MeOH 4/1 mixture and isolated in 34 and 42% yields. The chiral catalytic activity of their Mo(V) and Cu(II) complexes was studied in the asymmetric oxidation of aromatic sulfides.

Barr et al. [109] have utilized cesium salts of acrylic, methacrylic, and crotonic acid to react with Ts-β-CD in DMF. The reactions were done at elevated temperatures for one to three days. The products were purified by Sephadex column and isolated in yields from 29 to 76%. Products were tested to control the nitrile oxide cycloadditions regioselectivity by inclusion complexation of the reagent before the reaction.

Inoue et al. [110] reacted Ts-β-CD with the sodium salt of 4-aminohydrocinnamic acid in DMSO at 80 °C for 3 days. After precipitation from acetone, the product was purified by RPC and obtained in a 66% yield. The authors further reacted this compound with PEG carboxylic acid and investigated the PEG chain self-threading.

Gao et al. [111] have utilized sodium 4-aminobenzoate for the reaction. The reaction was in DMF and stirred for 3 days at 60 °C. The final product was isolated in a 51% yield after classical silica gel column chromatography. The β-CD derivative was further diazotized by *o*-carbaldehyde phenol to form azobenzene moiety. A light-powered [1]rotaxane consisted of this azobenzene modified CD and a Schiff base bridged by a metallosalen unit.

Maeda et al. [112] reacted Ts-β-CD with carboxyphenylacetylene sodium salt in DMSO at 80 °C for 46 h. After precipitation from acetone, the yield of the product was 59%. This monomeric unit was further polymerized into helical poly(phenylacetylene)s and their chiroptical properties were investigated.

Pedotti et al. [113] took sodium succinate and added it into the Ts-β-CD solution. The mixture was stirred for 24 h at 100 °C, and the final product was obtained after RPC purification. The yield was 45%. The authors formed a prodrug of the antiviral agent Acyclovir and investigated its release in acidic and neutral conditions and in the presence of porcine liver esterase.

Ueno et al. [114] let react 6^A^-*O*-(2-naphthalenesulfonate)-γ-CD with potassium ferrocene carboxylate in DMSO at 80 °C. After 3 h of stirring, the reaction was stopped, and the crude product was purified by recrystallization from the nBuOH/EtOH/water mixture. The product was isolated in a 24% yield. The authors further investigated its binding behavior in different solvent systems.

In their next paper [115], the authors prepared 9-anthracenecarbonyl-β- and γ-CD by reacting Ts-β-CD or 6^A^-*O*-(2-naphthalenesulfonate)-γ-CD with sodium 9-anthracene carboxylate in DMSO. Reactions were stirred for 6 h at 80 °C, and pure products were obtained in 20 to 36% yields after Sephadex column chromatography. Their self-association and host–guest complexation were investigated.

Some articles which do not fit into the above categories or describe interesting results follow. Jiao et al. [116] synthetized 6^A^-*O*-acyl-β-CDs derived from pentacyclic oleanane triterpenes by utilizing the previously mentioned (de)benzylation strategy and tested their biological activity and cytotoxicity. The authors prepared 6^A^-bromo-6^A^-deoxy-per-*O*-benzyl-β-CD, let it react with potassium oleanolic or echinocystic acid salts, isolated the products in 62 and 82%, and deprotected the remaining hydroxyl groups. This strategy ensures no formation of 3^A^,6^A^-anhydro-β-CD, a typical side-product in Ts-CDylations. Its yield depends on the basicity of the nucleophile, so with carboxylate is formed in a smaller amount.

Shipilov et al. [117] were also concerned about the regioselectivity of acylation reactions. The authors tested aromatic carboxylic acids (7 eq.), forming complexes with native β-CD. After the complex formation, the authors added a sulfuric acid (1.4 eq.), heated the reaction mixture in DMF to 130 °C, and stirred for 3 h. The reaction mixture was then neutralized by calcium hydroxide and purified by precipitation in diethyl ether. ^1^H NMR confirmed the formation of mono-substituted derivative, and regioselectivity was checked by ^13^C NMR. The authors prepared five different compounds in yields of around 80%.

Nielsen et al. [118] utilized 4-nitrophenyl esters of carboxylic acids instead of acyl chlorides. They investigated if the complexation of the 4-nitrophenyl ring into the CD cavity influences the degree of substitution and regioselectivity. The products were obtained in 50% to 80% yields after crystallization. Nevertheless, the effect of complexation on the reaction was negligible in the case of α- and γ-CD, even though the complex formation was proved and studied by ITC. For β-CD, the influence of the complexation on the reaction was more significant compared to the α-CD.

Martina et al. [119] tested their general procedure for the preparation of monoisostearoyl-α-, β-, and γ-CD to prepare and characterize their inclusion complexes with natural compound silibinin. To the solution of CD and TEA in DMF with the catalytical amount of DMAP, isostearoyl chloride DMF solution was added at −15 °C. Products were purified by RPC, and the most important fact is that this procedure could separate even different regioisomers. In the case of α-CD, 6^A^-*O*-regioisomer was obtained in a 40% yield and 2^A^-*O*- isomer in a 13% yield. For β-CD, both formed regioisomers 6^A^-*O*- and 2^A^-*O*- could be separated, and the yields were 25 and 10%, respectively. Moreover, γ-CD 6^A^-*O*- and 2^A^-*O*-isostearoyl regioisomers were prepared and isolated in 16 and 7% yield, respectively.

However, the authors were not satisfied with these results and came up with a different strategy for improving regioselectivity. After the addition of Cu(II) ions, CD molecules formed dimers. These dimers resemble a sandwich structure in which cooper ion is bound to secondary sides of CD molecules. Due to that, only primary hydroxyl groups are accessible for the reaction with acyl chlorides. Authors obtained pure 6^A^-regioisomers in 17 to 31% yield after column chromatography or recrystallization, after reactions with benzoyl, cinnamoyl, and phenylacetyl chlorides.

To sum up, if a pure 6^A^-*O*-acyl-CD is needed, then the Ts-CDylation strategy should be used. Column chromatography might be necessary to purify desired products from 3^A^,6^A^-anhydro-CD side-products. Another option is the (de)benzylation strategy. If a high purity product is not needed, the higher-yielding acyl chloride or coupling strategies followed by precipitation can be used to avoid column chromatography. Table 4 describes a number of articles using the various strategies described herein, along with yields and purification methods.

### 1.5. 6^A^-S-Aryls/alkyls/acyls of CDs

**Scheme 5:**
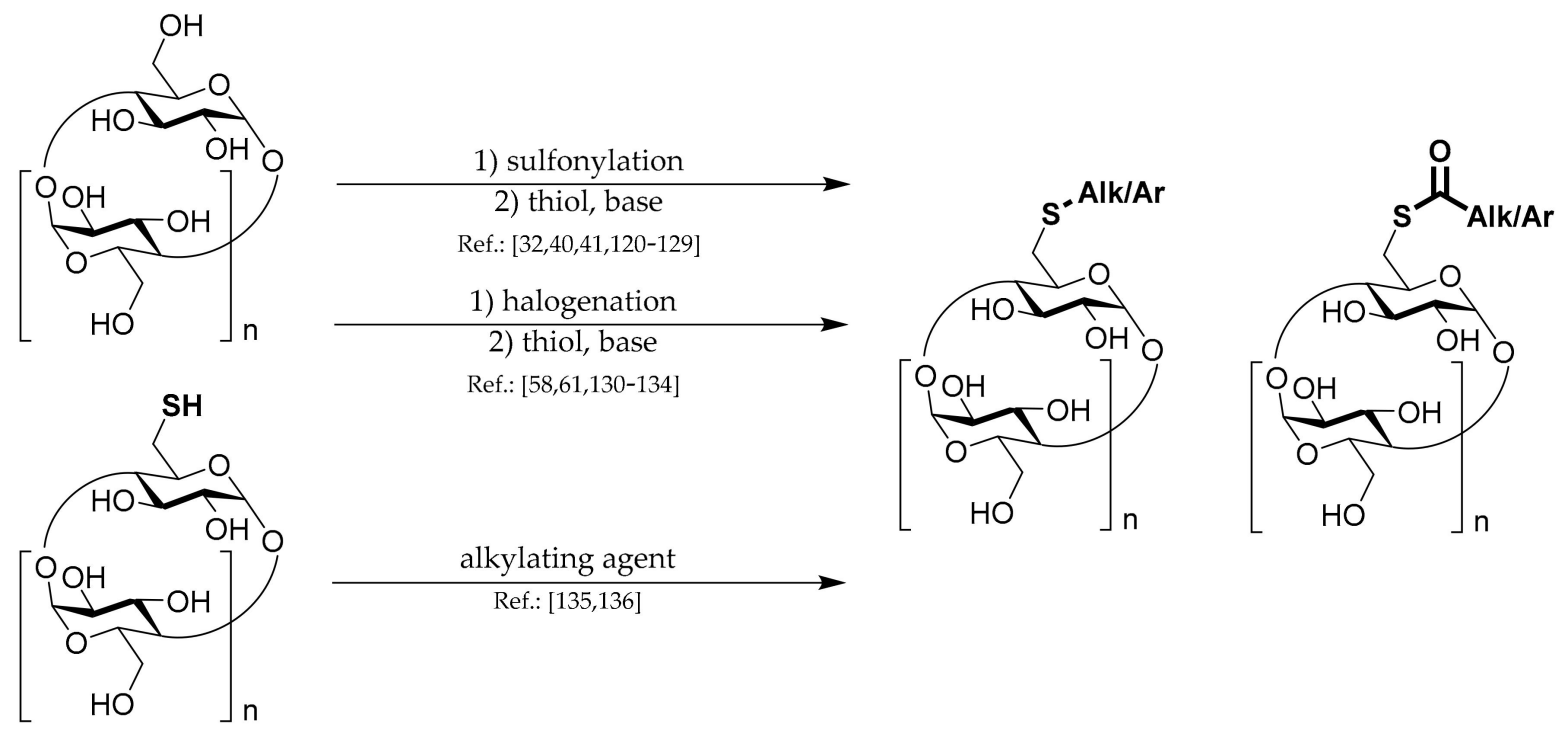
General methods for preparation of 6^A^-*S*-aryls/alkyls/acyls of CDs.

General methods for the preparation of these sulfur-containing CD derivatives are depicted in Scheme 5.

Concerning the synthesis of *S*-alkylated or acylated CD derivatives, the main strategy is Ts-CDylation of a salt of a thiolate with Ts-CD or in one case other alkylsulfonated CD.

Lo et al. [32] performed the reaction with 6^A^-(methanesulfonate)-α-CD. The reaction was done in DMF with potassium thioacetate at 50 °C and stirred for 12 h. The product was purified by RPC and obtained in a 64% yield. The next step, basic hydrolysis, was studied by NMR due to the significant change of CH_2_ signals in the CD molecule. According to these measurements, the reaction was finished in 45 min at room temperature.

Ekberg et al. [41] reacted Ts-β-CD with cysteamine hydrochloride in DMF/water mixture in the presence of ammonium bicarbonate. After 4 h of stirring at 60 °C, the product was purified via the Sephadex column and obtained in a 43% yield.

Defaye et al. [40] performed reactions of Ts-β-CD with peracetylated 1-thio-α-D-glucopyranoside sodium salt and β-anomer in 1,3-dimethyl-2-oxohexahydropyrimidine. Products were purified by preparative HPLC and isolated in yields around 60%.

Reetz et al. [120] reacted 2-(diphenylphosphino)ethanethiol with Ts-β-CD in a water/MeOH mixture. The pH was adjusted to 12 by sodium carbonate, and the reaction mixture was stirred for 2 days at 50 °C. The product was purified by recrystallization from a water/EtOH mixture. The yield was 66%. The compound was utilized as a bidentate ligand for the norbornadiene Rh(I) complex to combine molecular recognition and catalysis.

Peroche and Parrot-Lopez [121] synthesized perfluoroalkylated β-CD by reacting Ts-β-CD with 3-perfluorohexylpropanethiol in MeONa methanolic solution. The product was isolated by precipitation from acetone and EtOH in a 94% yield.

Milović et al. [122] reacted sodium 2-(2-mercaptomethyl)-propane-1-thiolate with Ts-β-CD in DMF. The reaction ran for 24 h at 60 °C. The product was purified by precipitation from water and isolated in a 31% yield. The product was utilized as a Pd(II) bidentate ligand. The potential of the formed complex to mimic peptidase was investigated.

Steffen et al. [123] synthesized a library of synthetic receptors for camptothecin. The general procedure for preparing their compounds was as follows: Ts-β-CD was dissolved in DMF, and TEA was added. After the addition of thiol, the mixture was stirred for 3 days at 60 °C. Products were purified by precipitation from acetone, and yields ranged from 32 to 95%.

Yuan et al. [124] have connected both cysteine enantiomers to a CD molecule. The reaction was done in a DMF/water mixture with sodium carbonate at 90 °C for 1.5 h. Both products were then purified by RPC and isolated in 80% yields. The cysteine-modified β-CD was further transformed into lactone by coupling the cysteine carboxylic group and CD hydroxyl. The direct correlation between lactone topology and cysteine enantiomer was observed. Lanza and Vecchio [125] also reacted cysteamine with Ts-β-CD in DMF with NaOH as a base. The reaction mixture was stirred for 7 h at 60 °C. The product was obtained in a 68% after Sephadex column purification. The product was subsequently transformed into salen-type ligands, and their Mn(III) complexes were formed. Their ability to mimic superoxide dismutases, catalases, and peroxidases was investigated.

Li et al. [126] mixed Ts-β-CD with 2-mercapto pyrimidine in DMF with potassium carbonate as a base. After 72 h of stirring at 80 °C, the product was purified by Sephadex column chromatography. The yield was 20%. Its self-assembly behavior was measured in both solution and the solid state and revealed pyrimidine-cavity inclusion and helical columnar superstructure.

Boonleang and Stobaugh [127] synthesized a negatively charged β-CD by the reaction of Ts-β-CD with 2-mercaptoethanesulfonate in DMF with NaOMe. The mixture was stirred for 2 h at 70 °C, and the final product was obtained by ultrafiltration in an 85% yield. The product was tested as a chiral selector in capillary electrophoresis.

Lampropoulou et al. [128] also synthesized a negatively charged β-CD. The authors utilized 3-mercaptopropanoic acid in DMF with NaH as a base. After 2 h of stirring at 100 °C, the product was purified by dialysis and isolated in a 98% yield. Mannose or *N*-acetylglucosamine were further connected via amide bond, and their bindings with ampicillin and lectins were investigated.

We prepared the series of 6^A^-(ω-sulfanyl-alkylene-sulfanyl)-β-CD derivatives [129] from Ts-β-CD and oligoethylene glycol dithiols in the MeOH/water mixture with sodium carbonate as a base. The properly degassed mixtures were stirred for 20 h at 50 °C, and products were purified by classical silica gel column chromatography. Yields were in the range of 60 to 88%. In addition, we described a method for simple conversion of CD disulfides, typical by-products encountered in syntheses of thiols, back to the desired products.

Another group of authors utilized 6^A^-deoxy-6^A^-halogens of CDs (X-CD). Kumprecht et al. [58] used Br-α-CD for this transformation. The reaction was done in DMF; after the proper degassing procedure, including vacuum-argon cycles, potassium thioacetate (1.2 eq.) in degassed DMF was added. After 12 h of stirring at rt, the product was precipitated and purified by RPC. The yield was 89%. The S-acetyl group was then hydrolyzed, and the 6^A^-thio-CD derivative was obtained by RPC in an 80% yield.

Ikeda et al. [130] used I-α-CD and let it react with sodium thioacetate in DMF. The reaction was stirred for 72 h at 80 °C and precipitated from acetone in the end. The S-acetyl group was then hydrolyzed in 1M NaOH for 1 h at 0 °C, and the product was purified via series of ion exchangers. The final yield was 36%. The compound was further transformed into a series of histidine-tethered dimers, and their ability to function as artificial hydrolases was investigated.

Rezac and Breslow [131] connected a cobalamin derivative to β-CD. 2-Nitrophenyldisulfido group in combination with tributylphosphine was used as a source of thiolate for the reaction with I-β-CD. The final product was purified by two RPC and isolated in a 20% yield. Cobalamin was covalently attached to β-CD, using this method, and the product was studied as an enzyme mimic.

Zhang and Breslow [132] performed the reaction with 5,5’-bis-acetylsulfanyl-2,2’-bipyridine in an ammonia/MeOH mixture. In this mixture, acetyls are cleaved, and thiolates are formed. After evaporation, I-β-CD in DMF was added, and the mixture was stirred for 3 h at 65 °C. The resulting dimer was purified by RPC, obtained in a 46% yield, and studied as an esterase mimic.

Zhang et al. [133,134] published the preparation of tetrathiafulvalene (TTF) β-CD derivatives in their two papers. The first one described the preparation of monosubstituted β-CD; the ability of β-CD to solubilize and stabilize the TTF-based radical cation was then demonstrated. The second paper was focused on the preparation of TTF-bridged β-CD dimer, and its interaction with porphyrin was studied. The synthetic protocols were similar, two different 2-cyanoethylsulfanyl-tetrathiafulvalene derivatives were dissolved in DMF, degassed, and cesium carbonate was added. Later, a DMF solution of I-β-CD was added, and the mixtures were stirred for 2 days at 80 °C. The final compounds were purified by MPLC or Sephadex column chromatography. Yields were 49% for monomer and 20% for the dimer.

Rodríguez-Lavado et al. [61] connected I-β-CD with Boc-protected cysteamine. The reaction was done in DMF with cesium carbonate as a base for 3 h at 75 °C. The product was purified by classical silica gel column chromatography and isolated in a 67% yield.

A few authors decided to use a 6^A^-thio-β-CD (HS-β-CD) to prepare *S*-alkylated or acylated β-CD derivatives. At first, the HS-β-CD had to be prepared. Fujita et al. [135] reacted Ts-β-CD with thiourea in MeOH/water mixture under reflux for 2 days. After colling down, 10% NaOH water solution was added, and the mixture was stirred for 5 h at 50 °C. The mixture was acidified, and trichloroethylene was added. The resulting precipitate was filtered and evaporated, and the product was recrystallized from water. The yield was 58%. HS-β-CD was further modified with *p*-hydroxy-*m*-nitrophenyl, and its complexation behavior with series of organic guests was studied by NMR and circular dichroism.

Choi et al. [136] utilized a different strategy. I-β-CD was dissolved in DMF, sodium sulfide (0.5 eq.) was added, and the mixture was heated to 80 °C and stirred for 15 h. The in situ formed sodium salt of HS-β-CD reacted with the remaining I-β-CD. The resulting dimer was purified by size-exclusion chromatography and isolated in a 48% yield. The dimer was utilized to enhance the bioavailability of β-naphthoflavone by supramolecular complexation.

In the end, we have to mention articles describing not so common but interesting strategies how to prepare these types of compounds. Cottaz and Driguez [137] prepared per-*O*-acetyl-(I-β-CD). This hydroxy-protected derivative reacted at rt with both anomers of peracetylated 1-thio-α-D-glucopyranoside. The reactions utilized in situ *S*-deacetylation and activation by cysteamine in HMPT in the presence of dithioerythritol. Both products were purified by classical silica gel column chromatography and isolated in yields 82% for α-anomer and 63% for β-anomer. The authors suggested their procedure as a general high-yielding method for the preparation of oligosaccharides branched CDs.

Sallas et al. [138] tested Mitsunobu reaction conditions. They obtained mono-substituted products by combining native β-CD, triphenylphosphine, diisopropyl azodicarboxylate, and various thiols. The reactions were performed in pyridine in yields ranging from 8 to 28% after RPC purification.

Jicsinszky et al. [139] used the ball milling technique to prepare various *S*-alkylated derivatives starting from Ts-β-CD. Reactions were tested with dodecanethiol, 3-mercaptopropionic acid, and thiourea. Dry and wet conditions were tested. With optimized procedures, products could be purified by ion-exchange columns, precipitation, and recrystallization. Yields were ranging from 23 to 73%.

To sum up this chapter, the most common and utilized method for preparing 6^A^-S-alkyl(aryl)/acyl-CDs is the Ts-CDylation with a proper thiol, deprotonated by bases such as NaOMe, CsOH, TEA, Na_2_CO_3_, or K_2_CO_3_. Chromatography methods are necessary to get pure products; precipitations are usually not sufficient. Another possibility, but not so frequent, is to change 6^A^-*O*-(aryl/alkylsulfonate)-CD for 6^A^-deoxy-6^A^-halogeno-CD first. A barely utilized option is to prepare HS-CD and let it react with alkyl halides and Michael acceptors. Table 5 shows the frequency of using the various strategies described herein, along with yields and purification methods.

### 1.6. 6^A^-Deoxy-6^A^-azides of CDs

**Scheme 6:**
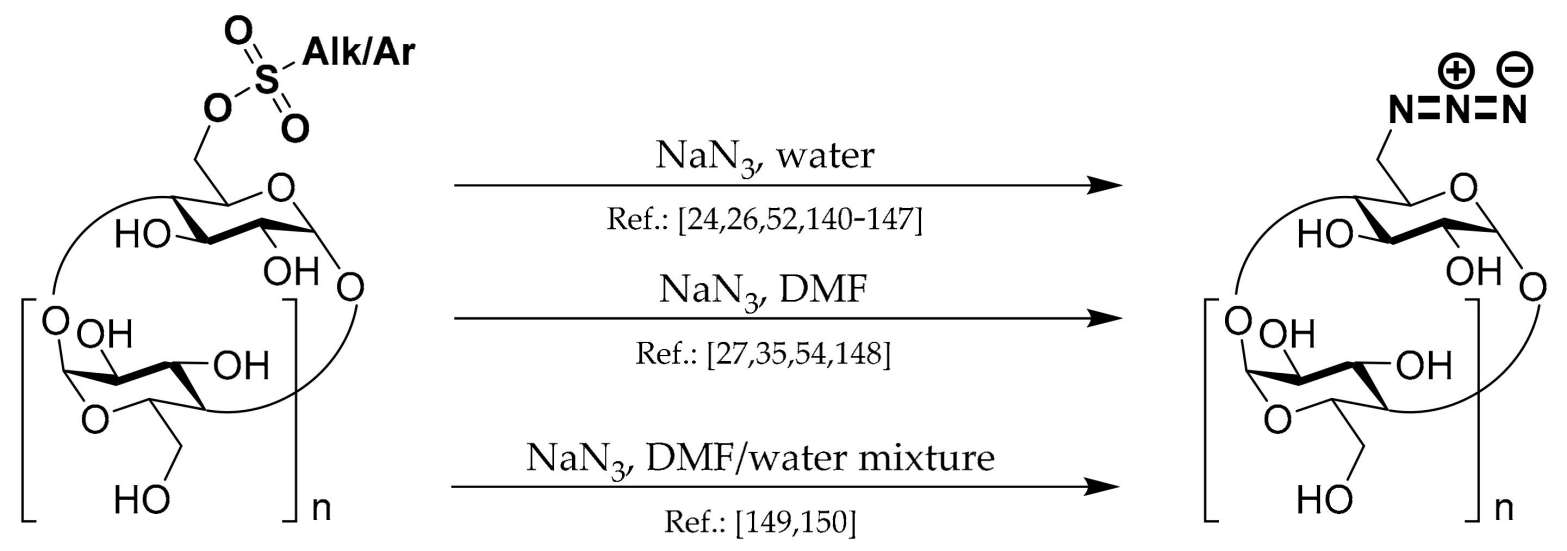
General methods for the preparation of 6^A^-deoxy-6^A^-azides of CDs.

Synthetic strategies leading to this type of CD derivatives can be divided into several groups which are depicted in Scheme 6.

Undoubtedly, Ts-CDylation of sodium azide with Ts-CD is the most utilized method used in the 6^A^-azido-6^A^-deoxy-CD (N_3_-CD) preparation. Some authors performed the reaction in *water*. Melton and Slessor [24] utilized 17 eq. of NaN_3_ in water. The reaction mixture was refluxed for 90 min. The purification was done by tetrachloroethane complex formation. The purified complex was destroyed by boiling in water, and the product was obtained in a 65% yield.

Samal and Geckeler [140] lowered NaN_3_ to 10 eq. and performed the reaction in refluxing water. The product was isolated by precipitation from acetone and isolated in a 90% yield. N_3_-CD was further utilized for the synthesis of CD-fullerene derivatives.

Quan et al. [141] also tried to prepare the product using 10 eq. of NaN_3_ in water. The significant change compared to other authors was that the mixture was stirred at room temperature instead of heating. After 8 h, the product was precipitated from acetone and obtained in a 29% yield, a significant decline compared to previously mentioned authors. Nevertheless, the compound was further utilized, and a hetero α-CD-β-CD dimer able to form micelles was prepared. Its drug-targeting potential was investigated.

Hein et al. [142] went even further with the NaN_3_ amount and used only 2 eq. in water. After stirring for 12 h at 90 °C, the product was purified by repeated precipitation from acetone. The yield was 72%. N_3_-α-CD was further coupled with alendronate as a targeting moiety, and a novel bone-targeting polyrotaxane delivery system was prepared.

Seo et al. [143] utilized 2 eq. of NaN_3_ also. The water solution was refluxed for 12 h, and the final product was recrystallized from water several times. The product was obtained in a 58% yield. The authors prepared polyrotaxane and connected Arg-Gly-Asp tripeptide via click reaction. Its interaction with cell surface receptor integrin β_1_ was investigated.

Bonnet et al. [144] added 10 eq. of NaN_3_ to the water solution of Ts-β-CD and stirred the mixture for 5 h at 80 °C. The final product was precipitated from acetone and isolated in a 98% yield. N_3_-β-CD was reduced, and reductive amination reaction with various 6-oxogalactosides was tested.

Liu et al. [145] utilized the same strategy. However, the final product was purified by dialysis after the precipitation from acetone. Probably to separate the product from the last traces of salts. The yield was 80%. Alendronate was then connected via click reaction. The binding to hydroxyapatite, the main component of tooth enamel, and inclusion complex with dexamethasone were investigated.

Tang and Ng [26] published a very detailed protocol. Ts-β-CD was refluxed in water with 20 eq. of NaN_3_ overnight. After the water evaporation, tetrachloroethane was added. The resulted β-CD-tetrachloroethane complex was separated from the aqueous phase. The pure product was obtained by recrystallization from water in an 85% yield.

Lai et al. [146] utilized a lower amount of NaN_3_ (8 eq.) and stirred the water solution for 4 h at 80 °C. The authors purified the product by RPC and obtained a 71% yield. N_3_-β-CD was subsequently methylated, and the azido group was reduced. Triplet sensitizer salen Pt(II) complex was connected via amide bond and its aggregation, 9,10-diphenylanthracence dimer (triplet acceptor) complex formation and the triplet–triplet annihilation upconversion emission were studied.

Uchida et al. [147] performed the Ts-γ-CD azidation in water with 10 eq. of NaN_3_. The mixture was stirred for 3 h at 80 °C, and the product was precipitated from acetone and further purified by RPC. The product was isolated in an 81% yield. *p*-Borono-benzoic acid was connected via click reaction, and the product was utilized as a hybrid cross-linker of polyvinyl alcohol to form a polyrotaxane gel.

Van Guyse et al. [52] utilized water as a solvent, and 5 eq. NaN_3_. The authors are one of few who admitted that precipitation from acetone is not enough to purify the product if the starting compound was not adequately purified. The authors utilized Ts-γ-CD with 28% purity; the rests were native γ-CD and over-tosylated by-products. After precipitation from acetone, the product was obtained in a 77% yield with 21% purity.

Other authors prefer to perform the reaction in DMF instead of water. Chwalek et al. [27] were one of those. Ts-α-CD DMF solution was heated to 140 °C for 2 h with 10 eq. of NaN_3_. The solvent was evaporated, and the crude mixture was purified by classical silica gel column chromatography. The yield of the product was 93%.

Petter et al. [33] published a comparison of two synthetic strategies. At first, the authors dissolved Ts-β-CD in DMF, added 0.5 eq of KI and 10 eq. of NaN_3_, and heated the reaction mixture to 65 °C. After 24 h of stirring, the mixture was cooled down, and an ion exchanger was added to remove salts. The product was precipitated from acetone and isolated in an 88% yield. For comparison, I-β-CD was utilized. Surprisingly, only 3 eq. of NaN_3_ was added in this case. The rest of the protocol remained the same, and the product was isolated in a 90% yield.

Jicsinszky and Iványi [35] utilized only 1.1 eq. of NaN_3_ and stirred the reaction for 1 h at 110 °C. The final product was recrystallized from a water/acetone 1/10 mixture with a 99% yield.

Nielsen et al. [148] synthesized the product and purified it first by repeated precipitations from acetone until no signal of NaN_3_ was visible in the IR spectrum. Then, the last traces were separated from the product by dialysis. The yield was 84%. The authors used the product for β-CD-dextran polymer synthesis via click reaction. Polymers were characterized by NMR and size exclusion chromatography. The β-CD in the polymer was still accessible as proven by ITC.

Yang et al. [149] were among the few who decided to react in a DMF/water 6/1 mixture with 7 eq. of NaN_3_. The reaction mixture was stirred for 5 h at 80 °C, and the final compound was obtained by recrystallization from the water/acetone mixture in a 93% yield. Triphenylene unit was connected via click reaction, and column liquid crystal behavior was observed and investigated.

Xu et al. [150] also performed the azidation in a DMF/water mixture. The solution containing Ts-γ-CD and 1.2 eq. of NaN_3_ was stirred for 6 h at 80 °C. The mixture was evaporated, and the product was obtained after RPC in a 90% yield. N_3_-γ-CD was subsequently reduced, and 9,10-diphenylanthracene was connected via an amide bond to form a γ-CD dimer. Its ability to complex sensitizer and influence of anthracene emitter/annihilator was investigated.

Palin et al. [54] tested Trips-γ-CD instead of Ts derivative. The authors performed the reaction in DMF with 1.5 eq. NaN_3_. After 24 h of stirring at 80 °C, the product was precipitated from acetone and obtained in a 94% yield.

Now we have to mention articles not fitting into the main trend. Fredy et al. [57] utilized Cl-α-CD and performed the reaction in DMF with 10 eq. of NaN_3_ under microwave irradiation at 150 °C. After 1 h, the mixture was filtered and purified by flash column chromatography. The yield was 80%.

Hanessian et al. [151] prepared N_3_-CDs by Appel reaction in their paper concerning selective CDs modifications. CD was dissolved in DMF, and PPh_3_, CBr_4_, and NaN_3_ were added. The reaction mixture was then stirred at rt for 6 h. Products were purified and separated from over-reacted by-products by classical silica gel column chromatography and isolated in 15 to 22% yields.

Parrot-Lopez et al. [152] utilized lithium azide instead of sodium analog and stirred the water solution for 4 h at 90 °C. The product was isolated in an 89% yield after crystallization from water. The neurotropic peptide Leu-enkephalin was coupled with N_3_-β-CD and fully characterized.

Trotta et al. [44] have synthesized N_3_-β-CD from Ts-β-CD and 1.5 eq. of NaN_3_ using microwave irradiation. The mixture was irradiated for 2 min at 85 °C. The product was recrystallized from a water/acetone 9/1 mixture and isolated in a 75% yield.

Strickland and Batt [153] used absolute EtOH as a solvent instead of water or DMF. The heterogeneous mixture of Ts-β-CD and NaN_3_ was refluxed for 12 h. The mixture was purified by precipitation from acetone. The yield was 92%. The compound was further modified and deposited onto gold nanorods surface and utilized as a detector for carbendazim fungicide.

Jicsinszky et al. [139] performed the reaction of Ts-β-CD with 3 eq. of NaN_3_ in a ball mill. The mixture was milled for 60 min. After sieving and washing the reactor, fractions were combined and recrystallized from the water/acetone mixture. Yields of repeated reactions were varying from 60 to 90%. 

To sum up this chapter, the most common and utilized method for preparing 6^A^-azido-6^A^-deoxy-CDs is the reaction of Ts-CD with NaN_3_ in DMF or water at elevated temperatures. A wide range of used azide equivalents, 1.2 to 30, can be found in the literature. In our experience, the amount ranging from 1 to 2 equivalents is entirely sufficient. Concerning the purification, repeated precipitation from acetone is usually enough for the β-CD derivative. When a significant excess of NaN_3_ is used, it is better to use crystallization or a water/acetone mixture for precipitations. In the case of α- and γ-CD derivatives, purity could depend on the solvent used. Both Ts-α-CD and Ts-γ-CD tend to be hydrolyzed in water and wet solvents. Then, column chromatography purification is necessary. Table 6 shows the frequency of various strategies described herein, along with yields and purification methods.

### 1.7. 1,4-Substituted Triazoles of CDs

**Scheme 7:**
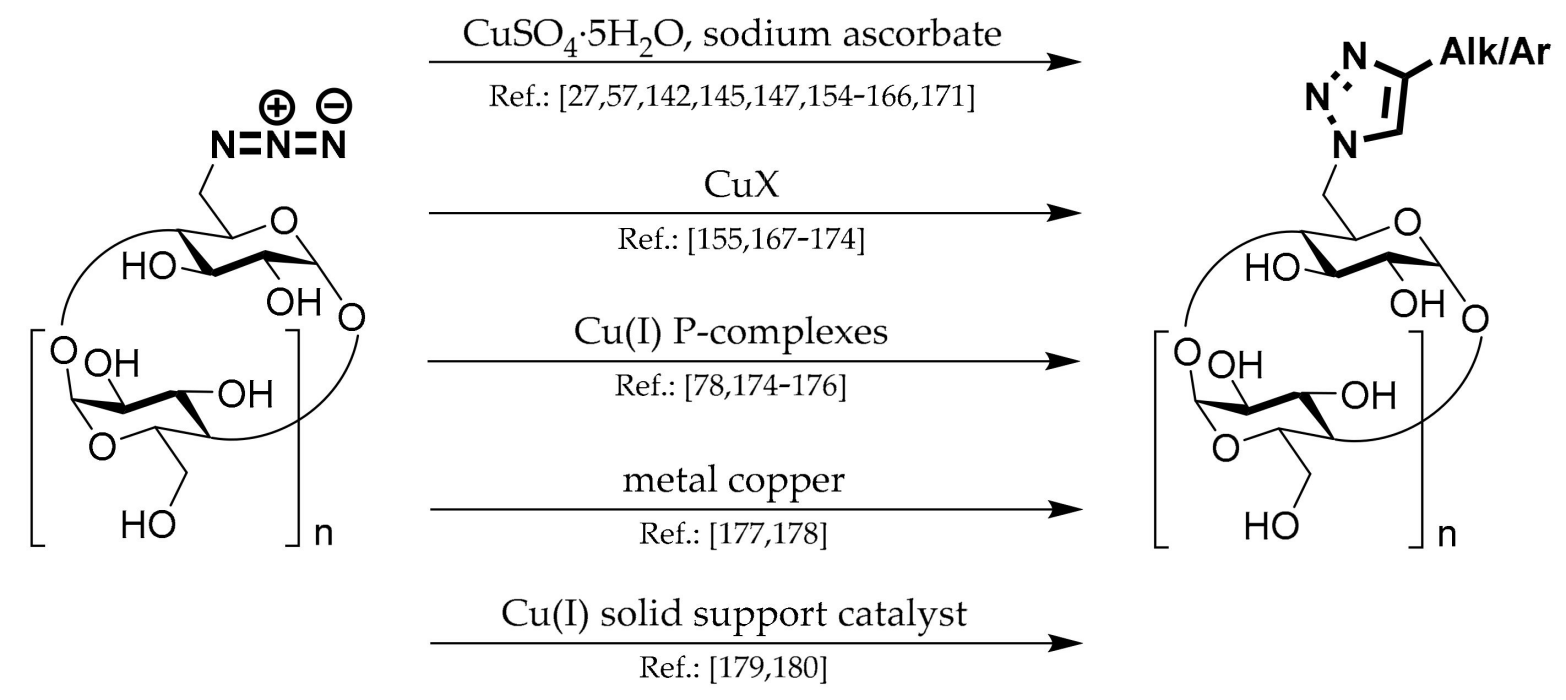
General methods for the preparation of 1,4-substituted triazoles of CDs.

This chapter describes products prepared from N_3_-CDs by copper-catalyzed azide-alkyne cycloaddition (CuAAC) reaction. Methods for their preparation can be divided by the Cu(I) system used for the reaction and are depicted in Scheme 7.

Most chemists work with copper sulfate pentahydrate (CuSO_4_·5H_2_O) and sodium ascorbate (NaASC) or ascorbic acid.

Chwalek et al. [27] performed the reaction with N_3_-α-CD and *N*-propargyl β-lactosylamide water/*i*PrOH 2/1 mixture. The authors added CuSO_4_ and NaASC in 0.2 eq. and 0.5 eq., respectively, and let the reaction run at rt for more than 12 h. The product was purified by column chromatography with MeCN/water mixture and isolated in a 75% yield. Xin et al. [154] prepared a series of dimers from bialkynyl-pillar[5]arene derivatives. The reaction was done in DMSO with 0.4 eq. of CuSO_4_ and 4 eq. of NaASC. Stirring at rt for 48 h and separation by HPLC gave the products yields from 30 to 50%. Fredy et al. [57] utilized slight modification of the general procedure to N_3_-α-CD with BODIPY and DOTA derivatives. The authors added PMDETA to stabilize formed Cu(I) ions. The reaction was performed in DMF, and the solution was stirred for 2 h at rt. Products were purified by flash column chromatography and obtained in yields of around 80%.

Bauer et al. [155] used CuI and PMDETA stabilizer (0.1 eq. of each) to connect cholesterylsuccinic acid propargylamide to α-CD. After stirring the DMF solution for 24 h at rt, the solvent was evaporated, and the crude product was suspended in phosphate buffer (pH 6.5), filtered, and washed. The yield of the product was 35%. They used the product for the construction of sliding anchored polymers and their insertion into phospholipid membranes.

Hein et al. [142] performed the reaction of N_3_-α-CD with acetylene-functionalized alendronate in the presence of tetrakis(MeCN)Cu(I)PF_6_ complex and TBTA as a stabilizer (0.1 eq. of each). The product was purified just by precipitation from EtOH and isolated in a 90% yield. However, the authors stated that the product was obtained in the form of a blue powder. This fact indicates Cu(II) impurities and that precipitation without further purification is insufficient.

Most publications deal, as usual, with β-CD derivatives, and the ascorbate method is the preferred one. Liu et al. [145] worked with acetylene-functionalized alendronate, as Hein et al. mentioned for α-CD analogs. However, in this case, the authors used a more common procedure for the synthesis. The reaction was done in water with 0.125 eq. of CuSO_4_ and 1.25 eq. of NaASC and stirred for 3 days at rt. The final product was purified just by precipitation from EtOH and obtained in an 82% yield.

Other authors also used water as a solvent for the CuAAC reaction, but the purification was more thorough. Gonsior and Ritter [156] synthesized positively charged β-CD by the reaction N_3_-β-CD with propargyl ammonium salt derived from methacrylate. After stirring with 0.05 eq. of CuSO_4_ and 0.1 eq. of NaASC at 100 °C for 12 h, pentaerythrit-tetrakis-(3-mercapto propionate) was added to chelate copper ions. The product was later precipitated from acetone and isolated in an 87% yield. The behavior of poly(pseudo-betaines) prepared from the polymerized product and adamantane carboxylate was then studied.

Zhang et al. [157] prepared the β-CD dimer by reacting with propargylamine-β-CD derivative at 70 °C for 24 h, with 2 eq. of CuSO_4_ and 5 eq. of NaASC. The product was purified by MPLC in a water/EtOH mixture and obtained in a 50% yield. Mori et al. [158] performed the reaction in a water/THF mixture and connected propargylated 1- or 2-naphthol derivatives with N_3_-β-CD. The reaction mixture, containing 1 eq. of CuSO_4_ and 3 eq. of NaASC, was stirred at 60 °C for 48 h. The mixture was precipitated from acetone, and the crude product was recrystallized from water/acetone 4/1 mixture. The yield was around 70%.

The largest group of authors performed these types of reactions in DMF, DMSO, or their water mixtures. Mourer et al. [159] synthesized β-CD dimers utilizing all three possible dipropargyloxybenzene regioisomers. The reaction was done in a water/DMF mixture at rt for 18 h with 0.4 eq. of CuSO_4_ and 1.7 eq. of NaASC. Products were purified by classical silica gel column chromatography in MeCN/water mixture, and yields of the products were around 60%. Diallo et al. [160] prepared ferrocene-β-CD by the reaction of ethynyl ferrocene and N_3_-β-CD in DMSO. The solution, containing 4 eq. of CuSO_4_ and 8 eq. of NaASC, was stirred at rt for 18 h. The crude reaction mixture was later stirred with ammonia solution to purify the product from copper impurities. In the end, the product was purified by column chromatography and isolated in a 60% yield. Watanabe et al. [161] performed click reactions with N_3_-β-CD, azido ferrocene derivative, and bis alkynylated naphthalene diimide derivative. All compounds were mixed in DMF, 1 eq. of CuSO_4_ and 4 eq. of NaASC, and 1 eq. of TBTA as a Cu(I) ions stabilized were added, and the mixture was stirred at rt for 50 h. The main product was isolated in a 10% yield by HPLC. They used the product as a probe for electrochemical DNA analysis.

Zhang et al. [162] reacted 2-ethynylpyridine with N_3_-β-CD in DMSO/water 1/1 mixture. To the solution, 0.1 eq. of CuSO_4_ and 0.2 eq. of NaASC were added, and the mixture was stirred at rt for 24 h. The product was precipitated from acetone and was washed with acetone until Cu was not detected by ICP-MS. The yield was 92%. Guo et al. [163] synthesized β-CD monomer, dimer, and trimer using mono-, di-, or tripropargyloxybenzaldehyde derivatives. Reactions were done in DMF with 1.1 eq. of CuSO_4_ and 2.4 eq. of NaASC at temperatures from 25 to 90 °C for 15 to 60 h. Products were purified by repeated recrystallizations from DMF/acetone mixture and obtained in yields around 80%. Sun et al. [164] connected ascorbic acid alkyne derivative with N_3_-β-CD in DMF with 0.05 eq. of CuSO_4_ and 2 eq. of NaASC. After stirring at rt for 12 h, the mixture was precipitated from acetone, and the crude product was purified by dialysis against EDTA and water. The yield was 95%.

Some authors utilize CuSO_4_ and NaASC or ascorbic acid in combination with microwave irradiation instead of classical heating. Cravotto et al. [165] synthesized homo and hetero CD dimer by the reaction of N_3_-β-CD and 2^A^-*O* propargylated α-, β-, and γ-CD derivatives. To investigate new strategies, the authors performed the reaction in a microwave reactor. The tBuOH/water 1/1 solutions containing, besides the CDs, 0.6 eq. of CuSO_4_ and 1.2 eq. of ascorbic acid were irradiated at 90 °C. The copper ions were complexed by DTPA sodium salt, and final products were obtained by precipitation from acetone. Yields ranged from 40 to 60%. Legros et al. [166] coupled alkynylated glycerol derivatives with N_3_-β-CD in DMSO/water mixture in the presence of 1 eq. of CuSO_4_ and 2 eq. of NaASC. The authors tested both microwave irradiation and classical heating in the oil bath. The temperature was held at 85 °C in both cases, but the time necessary for the reaction to complete was significantly different. Under microwave irradiation, the reaction was finished within 80 min, but in oil bath heating, 24 h were needed. Yields were in the range of 14 to 40%. NMR conformational analysis of the prepared dimers proved tumbling of one of the CD glucopyranose units.

Only one article describing the preparation of γ-CD derivatives from N_3_-γ-CD by CuAAC can be found. Uchida et al. [147] synthesized boronic acid-appended γ-CD as a cross-linker of polyvinyl alcohol chains to form a hydrogel. This γ-CD derivative was formed by the reaction of N_3_-γ-CD with propargyl ester of 4-borono-benzoic acid. The reaction was done water/DMF 1/1 mixture, and the catalytic system consisted of 0.05 eq. of CuSO_4_, 2 eq. of NaASC, and 0.05 eq. of TBTA stabilizer. After 6 h of stirring at rt, the reaction mixture was precipitated from acetone, purified by column chromatography using selective boron ion-exchange resin (Amberlite IRA743) and RPC. The yield of the product was 68%.

Another heavily utilized Cu(I) source is copper bromide (CuBr). Yan et al. [167] prepared β-CD monomer suitable for radical polymerization by reacting derivatized ethylene glycol ester of 4-pentynoic acid with N_3_-β-CD. Compounds were dissolved in DMF, 1 eq. of CuBr and 1 eq. of PMDETA stabilizer were added, and the mixture was stirred for 12 h at 35 °C. The crude product was filtered over alumina to remove copper ions; the final product was recrystallized from THF and obtained at a 92% yield. The product was used for the preparation of voltage-responsive vesicles based on two homopolymers end-capped with the CD and ferrocene derivatives. Cakir et al. [168] utilized Me_6_TREN (1 eq.) as a stabilizer for CuBr (1 eq.) for the reaction between propargylated d-mannose and N_3_-β-CD. The reaction was done in DMSO at 50 °C for 24 h. The product was isolated by precipitation from acetone and mixing with commercial copper chelating agent Cuprisorb. The yield was 67%; the interaction of the product with adamantane containing thermoresponsive copolymer was studied. Paolino et al. [169] used microwave irradiation and a mixture of 0.5 eq. of CuBr and a 0.5 eq. of DIPEA to combine propargylated quinoline-4-carboxamide derivative with N_3_-β-CD. After irradiating the DMF solution for 75 min at 85 °C, the product was purified by RPC in a water/EtOH mixture and isolated in a 30% yield. It was then tested as a component of hybrid drug delivery system, the second component being an adamantane modified dendrimer.

Copper iodide (CuI) is another favorite reagent for this type of reaction. Kim et al. [170] performed the reaction with ethynylestradiol derivative in DMF/MeOH 5/1 mixture with 5 eq. of CuI and 0.6 eq. of DIPEA. The reaction mixture was stirred for 24 h at rt, and then solvents were evaporated. The solid residue was dissolved in ammonia solution to complex copper ions, and the product was precipitated from acetic anhydride. The yield of the product was 64%. This estradiol-CD conjugate was proven to penetrate cellular but not a nuclear membrane. Tomanová et al. [171] decided to prepared flavin-CD conjugates and so utilized several alkynyl alloxazine derivatives. First, reactions were performed in the presence of CuI (0.03 eq.) and DIPEA (1.7 eq.) in DMF at rt for 24 h. Products were isolated in low to moderate yields after RPC. However, some compounds could not be prepared by this method, probably due to oxidation of Cu(I) to Cu(II) ions by alloxazine compounds. For this reason, the authors performed the reactions again, but in the presence of CuSO_4_ (0.1 eq.), NaASC (0.2 eq.), and TBTA (0.2 eq.) This time, yields of the products increased drastically. The prepared conjugates were studied as monooxygenase mimics in enantioselective sulfoxidations. Shi et al. [172] synthesized β-CD dimer based on BODIPY moiety. Bis-alkynylated BODIPY derivative was reacted with N_3_-β-CD in the DCM/EtOH/water 12/1/1 mixture in the presence of 0.2 eq. of CuSO_4_ and 0.35 eq. of NaASC. The reaction mixture was refluxed for 15 h, then 0.5 eq. of CuI was added, and the mixture was stirred for another 12 h. The product was purified by classical silica gel column chromatography in DCM/MeOH mixture and size exclusion chromatography. The yield of the product was 28%. Photoinduced processes of its complexes with tetrasulfonated porphyrins were then studied.

Chiba et al. [173] reacted alkynyl nucleoside derivative with N_3_-β-CD in water. The CuCl was added, and the mixture was stirred for 1 h at rt. The product was isolated in an 89% yield by RPC purification. Similar conjugate was prepared from adamantyl azide, and short DNA duplexes based on the complexation of these components were studied.

Another valuable and utilized group of reagents are copper(I) halide complexes with phosphorus-based compounds.

Casas-Solvas et al. [78] developed preparations of monomeric and dimeric β-CD compounds containing an azobenzene unit. They used the reaction of propargyloxy benzene or dipropargyloxy azobenzene derivatives with N_3_-β-CD in the presence of (EtO)_3_PCuI (0.2 eq.) in DMF. The solution was stirred for 2 h at 100 °C, and the products were purified and isolated by recrystallization from water or classical silica gel column chromatography in yields around 70%.

Legros et al. [174] utilized various reagents to connect alkyne-derivatized nucleobases to β-CD. The authors tested CuI (9 eq.) in tBuOH/water 1/1 mixture under microwave irradiation at 85 °C for 40 min, CuSO_4_ (0.5 eq.) and NaASC (1 eq.) in DMSO at rt for 12 h, or (EtO)_3_PCuI (0.5 eq.) in DMF at 90 °C for 48 h. In all cases, products were purified by semi-preparative HPLC. In terms of yields, no advantage of one reagent over the other two was observed. A set of β-CD-nucleobase conjugates was prepared as potential supramolecular elements.

Bai et al. [175] prepared an amphiphilic β-CD dimer that consists of one hydrophobic ibuprofen and two hydrophilic β-CD moieties. The reagent of choice was CuBr(PPh_3_)_3_ complex (0.1 eq.) The reaction was done in DMF and stirred for 18 h at 60 °C. The mixture was filtered over the alumina column to separate the product from copper ions and then precipitated from acetone. The yield of the dimer was 72%. The host–guest behavior of the dimer in various solvents was then studied.

Diget et al. [176] synthesized β-CD monomer derived from propargyl methacrylate, later used in radical polymerization and study of the copolymer properties. The reaction was performed in DMF with Cu(CH_3_CN)_4_PF_6_ (0.04 eq.) and TBTA (0.04 eq.) catalytic system. The product was purified by precipitation from acetone, stirring with Amberlite GT74 chelating resin, and dialysis against water. The monomer was isolated in a 92% yield.

Metal copper is another reagent that could be utilized for these cycloaddition reactions.

Cintas et al. [177] published general protocols for CuAAC using metal copper and ultrasound, one example being the formation of β-CD dimer derived from 1,3-bis(propargyloxy)benzene. The authors tested an ultrasound procedure or ultrasound together with microwave irradiation. Reactions were done in DMF or dioxane/water 4/1 mixture and 2 eq. of metal copper. Mixtures were sonicated or sonicated and irradiated for 2 h at 100 °C. In all cases, the product was isolated in yields around 70% by RPC purification. Rinaldi et al. [178] tested the ball-mill technique in combination with the metal copper. N_3_-β-CD and phenylacetylene were mixed with metal copper (1 eq.), placed in a milling jar, and stirred for 30 min at 650 rpm. The mixture was separated by RPC, and the product was obtained in an 81% yield.

The last part is devoted to solid-supported Cu(I) catalysts.

Cintas et al. [179] tested Cu(I)/C catalyst for the model reaction of phenylacetylene with N_3_-β-CD. The authors tested two synthetic strategies. The first could be called a one-step protocol. It was a reaction of Ts-β-CD with NaN_3_ in the presence of phenylacetylene and 0.1 eq. of Cu(I)/C in DMF or tBuOH/water 1/1 mixture. The solution was placed in a microwave reactor and irradiated at 85 °C for 20 min. The product was isolated in a 95% yield. The second one was a two-step protocol when N_3_-β-CD was prepared and purified and then combined with phenylacetylene and Cu(I)/C catalyst under the same conditions. In this case, the yield was 77%.

Megia-Fernandez et al. [180] published a paper about the preparation and utilization of Cu(I) modified silica gel. The authors prepared series of modified silica gel which differs by the nature of nitrogen compounds connected to the solid support, which can complex Cu(I) ion and catalyze CuAAC reactions or function as a chelating agent which can purify the reaction mixture from copper ions. Azide and acetylene modified silica gels were also prepared and tested as scavengers for azido and alkynyl reaction partners. The authors performed a considerable number of reactions with great results, and one of them concerned CD. The authors mixed N_3_-β-CD and alkyne modified glucose (2 eq.) together with CuCl in water. The mixture was irradiated in a microwave reactor for 20 min. Then, azido-modified silica gel was added, and the mixture was irradiated for another 30 min to eliminate the excess of the alkyne-modified glucose. The reaction mixture was filtered, and the content of copper was determined to be 45 ppm by AAS. The solution was filtered over chelating agent-modified silica gel, and the content of copper was below the limit of AAS. According to the authors, the product was isolated in a quantitative yield.

To sum up this significant chapter, there are many possibilities to perform the CuAAC reaction. The most common combination of reagents is CuSO_4_ and NaASC or ascorbic acid. The second most utilized Cu(I) source would be CuBr, but other copper halides could also be used. All solvents should be degassed adequately by bubbling with nitrogen or argon. Freeze-pump-thaw cycles could be utilized for this purpose also. If solvents are not adequately degassed and the reactions are not kept in an inert atmosphere, Cu(I) could be oxidized to Cu(II) ions. Then, more NaASC must be used for the CuSO_4_ system or a stabilizer such as TBTA or DIPEA for copper(I) halides. Concerning the purification, simple precipitation is usually insufficient, and copper ions could still be present in the product. Column chromatography, recrystallizations, stirring with amines, or the addition of solid chelators are often necessary. Table 7 shows the frequency of using the various strategies described herein, along with yields and purification methods.

### 1.8. 1,5-Substituted Triazoles of CDs

Surprisingly, only very few articles could be found describing the synthesis of these regioisomeric triazole CD derivatives. Liu et al. [181] studied the self-assembly behavior of the azobenzene modified β-CD derivatives. For this purpose, the authors prepared in- and out-isomers, with the azobenzene moiety situated in or out of the cavity, respectively, by two different strategies. The out-isomer was prepared from 4-propargyl azobenzene by reacting with N_3_-β-CD in the presence of CuSO_4_ (0.4 eq.) and NaASC (0.8 eq.), conditions leading to 1,4-substituted triazole. The reaction was done in a water/EtOH 2/1 mixture and stirred for 8 h at rt. The product was purified by precipitation from acetone and washing with ammonia solution. The yield was around 70%. The in-isomer was prepared by thermal Huisgen cycloaddition. The exact amount of 4-propargyl azobenzene was added into water/EtOH 2/1 solution of N_3_-β-CD, and the mixture was stirred for 3 days at 85 °C. Both 1,4- and 1,5-substituted triazole derivatives were formed. However, the desired 1,5-substituted regioisomer (in-isomer) could be recrystallized from water in a 69% yield.

The second paper which could be mentioned was published by Munteanu et al. [182]. The authors tested microwave irradiation conditions on the model reaction between propargyl methacrylate and N_3_-β-CD. The reaction was done in DMF with 0.01 eq. of CuSO_4_ and 0.02 eq. of NaASC. The mixture was irradiated at 100 °C for 30 min, and the product was isolated in an 84% yield by precipitation from acetone. The product contained only 1,4-triazole regioisomer. For a comparison, classical oil bath conditions were tested for the same reaction. After 30 min at 100 °C, only 57% of the 1,4-triazole product was isolated. The product was a mixture of both triazole regioisomers in a 3/1 ratio favoring 1,4 triazole if the reaction time was prolonged.

### 1.9. 6^A^-Amines and Aryl/alkylamines of CDs

#### 1.9.1. Primary Amine

**Scheme 8:**
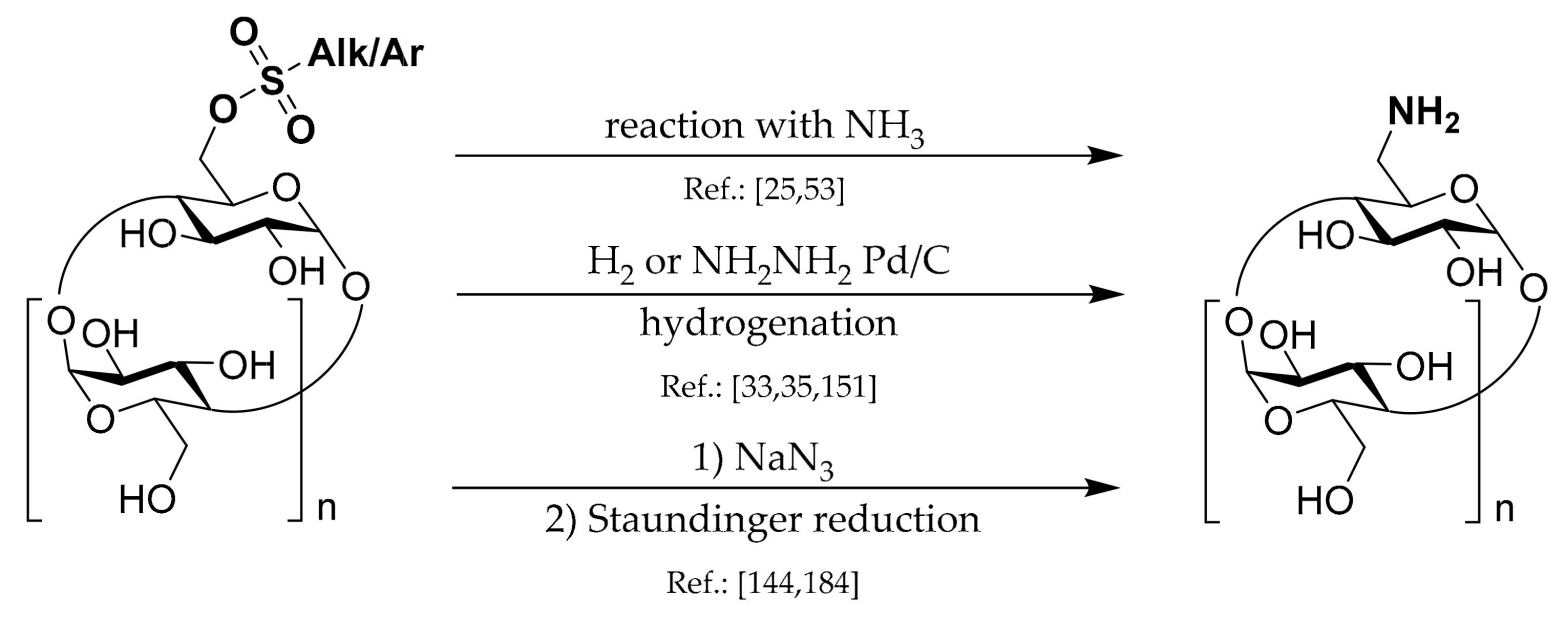
General methods for the preparation of 6^A^-deoxy-6^A^-amines of CDs.

There are several methods how to prepare 6^A^-amino-6^A^-deoxy-CDs (H_2_N-CDs) and the most common ones can be found in Scheme 8.

Brown et al. [25] tested the reaction of Ts-α-CD with a concentrated ammonia-water solution. The major product was native α-CD because of hydrolysis of the tosyl group. However, when the reaction was performed in dry DMF with condensed liquid ammonia at rt for 18 h, the product was formed. Purification was done by precipitation from acetone and proper washing of the solid product. The yield was 70%. The same protocol was also used for the preparation of H_2_N-β-CD. In this case, the product had to be purified by a strong cation exchanger. By-products were eluted by water and the product by ammonia solution. The yield was 54%.

Hanessian et al. [151] utilized a hydrogenation strategy that became popular over the years since their publication. N_3_-α-CD was hydrogenated in Parr hydrogenator (2 bar of H_2_) in the presence of a Pd/C catalyst. MeOH/water 1/1 solvent mixture was used, and at the end of the reaction, the mixture was filtered and evaporated. The product was obtained in a 90% yield.

Yuan et al. [183] utilized the Mitsunobu reaction to modify α-CD with phthalimide. Standard DEAD and PPh_3_ reagents were used, and after 5 h of stirring at rt, the reaction mixture in DMF was precipitated from acetone. The product was purified by RPC. Mono-substituted product was isolated in a 41% yield. This compound was further dissolved in water, and an excess of hydrazine hydrate (100 eq.) was added. The solution was stirred at 60 °C for 12 h. The product was purified by ion-exchange chromatography and isolated in an 88% yield.

Tang et al. [184] applied Staudinger reduction to obtain H_2_N-α-CD and H_2_N-γ-CD. The corresponding N_3_-CD was mixed with PPh_3_ in DMF, and the solution was stirred for 2 h at rt. Then water was added, and the mixture was reflux for another 3 h. The products were isolated in 94% and 90% yields after precipitation from acetone. Their ability for chiral resolution of anionic racemates was then evaluated.

Most authors used the Staudinger reduction. Bonnet et al. [144] published the first complete protocol for β-CD. N_3_-β-CD was mixed with PPh_3_ (1.7 eq.) in DMF. Aqueous NH_3_ (24 eq.) were added, and the reaction mixture was stirred for 4 h at rt. The product was precipitated from acetone and isolated in a 98% yield.

Another frequently utilized strategy is hydrogenation. Petter et al. [33] published this procedure at the beginning of the 90s for β-CD. N_3_-β-CD was dissolved in water, and Pd/C was added. A balloon filled with hydrogen was connected, and the mixture was stirred for 12 h at rt. After filtration, evaporation, and drying, the product was obtained in an 87% yield.

Reddy et al. [185] published a method based on metal indium and ammonium chloride. N_3_-β-CD was dissolved in DMF/MeOH 4/1 mixture, and indium together with ammonium chloride (1.2 eq. for both) was added. The reaction mixture was stirred for 3 h at 80 °C. After filtration, the mixture was poured into water, and the precipitated product was collected and dried. The yield was 94%.

Jicsinszky and Iványi [35] published the strategy which should be mentioned. N_3_-β-CD was suspended in absolute MeOH, cooled to -25 °C, and palladium on charcoal suspended in water was added. After warming to rt, the hydrazine hydrate (5 eq.) was added as a hydrogen donor. The heterogeneous mixture was refluxed for 20 min. Then, the product was purified by filtration, acidification, and recrystallization from MeOH. The product was isolated in a 90% yield.

Pham et al. [53] have prepared H_2_N-γ-CD as a starting compound for the synthesis of CD dimers. Ts-γ-CD was dissolved in 28% aqueous NH_3_ solution and stirred for 5 days at rt. The product was then purified by ion-exchange column chromatography. The yield of the product was only 18%, probably due to hydrolysis of the tosyl group and formation of 3,6-anhydro-γ-CD.

#### 1.9.2. Secondary Amines

**Scheme 9:**
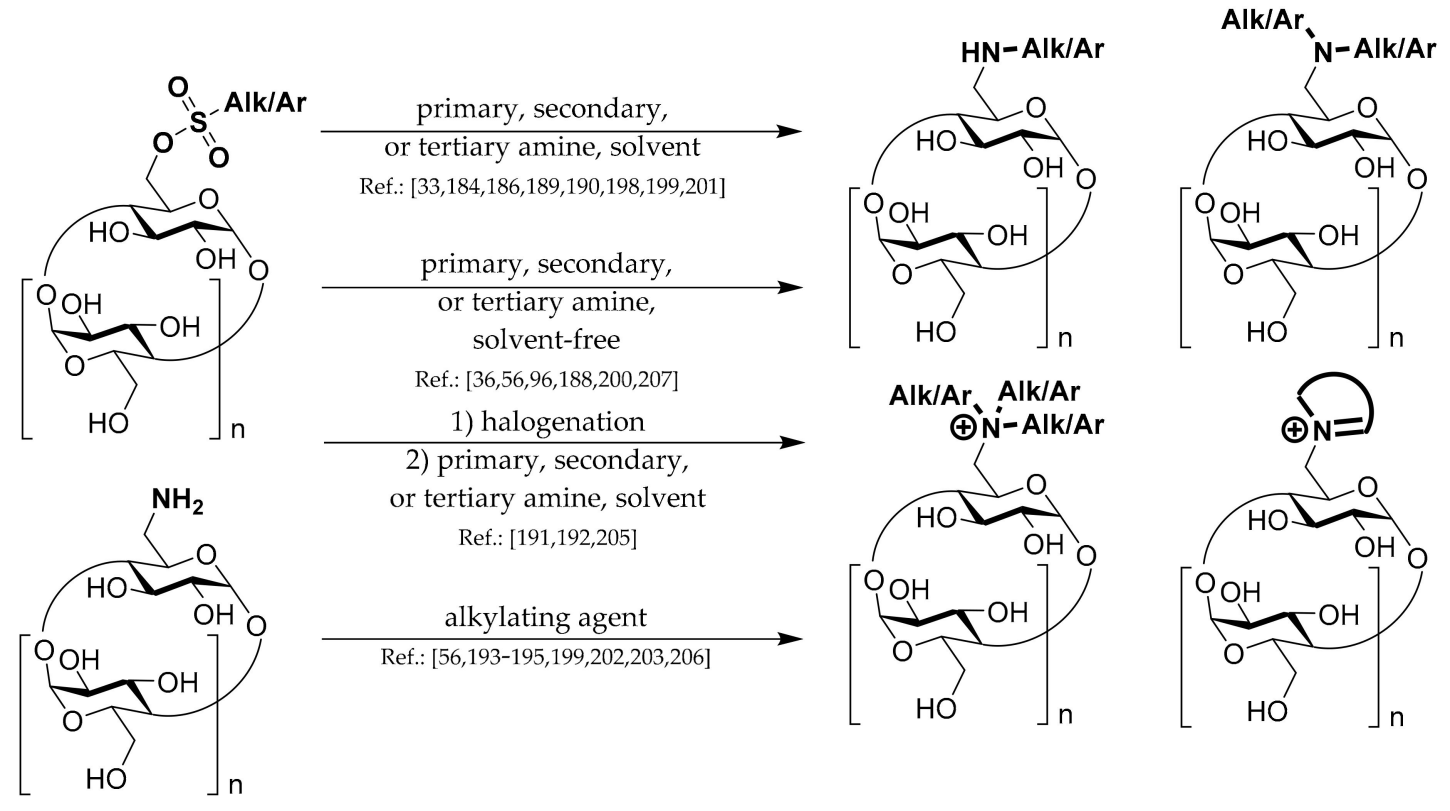
General methods for preparation of 6^A^-aryl/alkylamines of CDs.

General methods for the synthesis of 6^A^-aryl/alkylamines of CDs, including secondary, tertiary, and quaternary amino derivatives are depicted in Scheme 9.

The nucleophilic substitution reaction of Ts-CD, eventually X-CD, by appropriate primary amine was utilized in most published procedures.

For α-CD, Onagi et al. [186] performed this reaction in *N*-methyl pyrrolidone with diaminostilbene derivative in the presence of KI to speed up the reaction. The mixture was heated to 80 °C and stirred for 40 h at this temperature. The product was purified by HPLC, isolated in a 19% yield, and used for the preparation of dimeric “hermaphrodite” [2]rotaxane.

To prepare the secondary amines of all three common CDs, we [56] utilized solvent-free conditions and dissolved the corresponding Ts-CDs in liquid amines. Mixtures were stirred for 18 h at 60 °C, and strong cation-exchange resins purified products with yields of around 70%. The products were used to prepare permanently positively charged CD derivatives which were then attached by a strong electrostatic bond to the Nafion carrier [187].

Most authors used the solvent-free strategy. Gao et al. [96] dissolved Ts-β-CD in ethanolamine and stirred the mixture for 20 h at 110 °C. The product was precipitated from acetone and obtained in 88% yield after drying. Next to the standard oil bath heating, microwave irradiation was also described. Puglisi et al. [188] synthesized a series of secondary amine CD derivatives to test the generality of the developed method. Ts-β-CD was dissolved in appropriate primary amine (150 eq.), and the solution was irradiated in a microwave reactor at 85 °C for 30 min. Products were purified by RPC and obtained in yields ranging from 60 to 95%.

The second most common method also uses Ts-β-CD; however, a solvent is present next to a reagent, mainly due to the solid nature of appropriate amines. Petter et al. [33] dissolved β-CD derivative and octylamine or hexadecylamine (2 eq. both) in DMF. The solution was heated to 70 °C, DMAP (2 eq.) and KI (0.2 or 0.5 eq.) were added, and heating continued for 30 h. Products were purified by a series of column chromatographies, including ion-exchange column, and isolated in 7 and 25% yields. The cooperative binding of the prepared mono-6-(alkylamino)-β-CDs was then studied.

Liu P. et al. [189] utilized NMP instead of DMF. The reaction with 5 eq. of (2*S*,3*S*)-2,3-*O*-isopropylidene-l,4-tetramethylenediamine in the presence of KI (0.15 eq.) was done at 75 °C for 8 h. The product was purified by ion-exchange column chromatography, obtained in a 74% yield, and evaluated as a chiral selector in capillary electrophoresis.

Liu Y. et al. [190] prepared L-tryptophan-modified CD by reacting Ts-β-CD with L-tryptophan in a water triethanolamine mixture. The mixture was refluxed for 24 h, and the final product was obtained after several column chromatographies in a 21% yield. Its molecular recognition and enantioselectivity to aliphatic alcohols were then evaluated.

Some authors make use of I-β-CD. Ueno et al. [191] utilized it for the reaction with naphthalene derivatized amine in DMF. The amine was in a significant excess (80 eq.), and the solution was heated to 80 °C and stirred for 24 h. The product was precipitated from acetone and purified by Sephadex column chromatography. The yield was 23%. The excimer formation in inclusion complexes of this host was then studied. Lo Meo et al. [192] synthesized a series of amino CD derivatives by their reaction with I-β-CD in pyridine. Amine (5 eq.) was added to the β-CD solution, and the mixture was stirred at 60 °C for 18 h. Final amino CD derivatives were purified by classical silica gel column chromatography and obtained in yields ranging from 60 to 80%. Their binding constants with nitrobenzene derivatives were then determined spectrophotometrically.

The third-largest group of authors switched the reagents’ roles and used H_2_N-β-CD, utilizing CD as a nucleophilic partner in a reaction. There are several possibilities for how to make use of this compound. Kikuchi et al. [193] prepared β-CD trimer by the nucleophilic substitution reaction of 2 molecules of H_2_N-β-CD with 6^A^, 6^D^-dideoxy-6^A^, 6^D^-diiodo-β-CD in DMF. The mixture was heated at 80 °C and stirred for 12 h. The trimer was purified by Sephadex column chromatography and obtained in a 13% yield. Deng et al. [194] performed a nucleophilic aromatic substitution reaction with sodium 2,4,6-trinitrobenzene-1-sulfonate in water. The solution was stirred for 1 day at rt; the product was precipitated from acetone and isolated in a 75% yield. The prepared guest-modified CDs were then used for the preparation of supramolecular hydrogels. Prashar et al. [195] modified β-CD with squaric acid by Michael addition/elimination reaction of H_2_N-β-CD with series of squaric acid esters derivatives. Reactions were done in a water/DMF 5/1 mixture, and after 2 h of stirring at rt, the mixture was precipitated from acetone. Products were obtained in yields of around 60% and used to modify proteins to prevent their aggregation. Bonnet et al. [144] performed a reductive amination reaction with D-galacto-hexodialdo-1,5-pyranoside derivatives. Reactions were done in DMSO and 5 eq. of pyranoside together with 50 eq. of NaBH_3_CN was added. Mixtures were stirred for 6 h at 60 °C. Products were purified by preparative RP-HPLC and isolated in yields ranging from 20 to 70%.

For reductive elimination, opposite roles of reactants are also possible. Yoon et al. [196], in their article about the general method for the synthesis of cyclodextrin aldehydes, described the reaction of 6^A^-deoxy-6^A^-oxo-β-CD with aniline. CD was dissolved in water; aniline (12 eq.) and NaBH_3_CN (2.5 eq.) were added, and the mixture was stirred for 6 days at rt. The product was purified by precipitation from acetone and obtained in an 88% yield. The authors stated that if it is necessary, the product could be further purified by RPC.

In the end, one last article does not fit into previously described categories. Sallas et al. [197] synthesized β-CD dimer by applying Mitsunobu reaction conditions. β-CD and PPh_3_ (12 eq.) were added to *N,N’*-Bis(2-aminoethyl)-1,10-phenanthroline-2,9-dicarboximide (6 eq.) in DMF; the iPrAD (12 eq.) was added, and the mixture was stirred for 3 h at rt. The mixture was precipitated from acetone, and the product was isolated by revere-phase column chromatography in a 2% yield.

For γ-CD, only the strategies described already for α- and β-CD were used. Nakamura and Inoue [198] synthesized a 2-(dimethylamino)ethylamine CD derivative and tested its ability to enantiodifferentiate photocyclodimerization of 2-anthracenecarboxylate by electrostatic interactions and complexation. They reacted Ts-γ-CD with 2-(dimethylamino)ethylamine (5 eq.) and KI (0.1 eq.) in NMP for 8 h at 70 °C. The product was then precipitated from EtOH, further purified by ion-exchange column chromatography, and obtained in a 39% yield. De los Reyes-Berbel et al. [199] developed a synthesis of a complex γ-CD derivative that behaves like a ”turn off-on” biosensor. The initial γ-CD intermediate was prepared from Ts-γ-CD with ethanolamine (20 eq.) in DMF. The reaction mixture was irradiated at 500 W at 80 °C for 1 h. The product was isolated and purified by precipitation from acetone in the yield of 88%.

Suzuki et al. [200] used solvent-free conditions to prepare an amino-γ-CD derivative, later modified by pyrene moiety and tested as a supramolecular probe for bicarbonate. Trips-γ-CD was dissolved in *N*,*N*-bis(3-aminopropyl)amine and stirred at 40 °C for 3 h. The product was isolated in a 37% yield by precipitation from acetone.

#### 1.9.3. Tertiary Amines

The most used strategy for preparing CD tertiary amines is the reaction of Ts-β-CD or I-β-CD with a secondary amine in a solvent. In all published procedures, DMF was utilized to solubilize β-CD and amine. Rosenthal and Czarnik [201] prepared β-CD-cyclen conjugate to test its ability to catalyze transacylation reactions. Ts-β-CD and cyclen (4 eq.) were dissolved in DMF and heated to 90 °C for 24 h. The product was precipitated from EtOH and further purified by ion-exchange column chromatography. The conjugate was obtained in a 49% yield.

The second most utilized strategy is similar to the first one but under solvent-free conditions. Hacket et al. [36] synthesized a series of amine derivatized β-CDs and tested their peptide complexation capabilities. Ts-β-CD was dissolved in 1-methylpiperazine and stirred at 70 °C for 15 h. After evaporating the amine, the product was precipitated from EtOH and isolated after ion-exchange column chromatography in a 75% yield. Puglisi et al. [188], already mentioned in the secondary amines section, used their microwave irradiation protocol on β-CD tertiary amine synthesis with morpholine and diethanolamine. In both cases, yields were higher than 95% after RPC. Using the solvent-free strategy we used for secondary amines [56], we also prepared tertiary CD amines from secondary liquid amines *N,N,N´-*trimethylethane-1,2-diamine and *N,N,N´*-trimethylpropane-1,3-diamine. Only 30 eq. of amines was enough to solubilize the starting compound, and solutions were stirred for 12 h at 70 °C. Again, products were purified by strong cation-exchange resins and isolated in 69 and 86% yields.

The third strategy is the alkylation, Michael addition, or condensation reaction of CD secondary amine. Rong et al. [202] used this strategy in their work focusing on artificial redox enzymes. *O*-Phenylenediamine-derived β-CD reacted with alloxan to form *N*-isoalloxazinomethyl moiety. Alloxan in aqueous HCl solution was mixed with β-CD derivative water solution at 78 °C, stirred for 15 min, and poured into acetone. The product was further purified by preparative HPLC and isolated in a 25% yield. Surprisingly, almost all published γ-CD tertiary amines were prepared using the strategy. Hamada et al. [203] synthesized naphthalene derived double-armed γ-CD and studied its self-complexation. H_2_N-γ-CD was alkylated by iodo ethylene glycol naphthalene derivative (8 eq.) in DMF, in the presence of K_2_CO_3_ (1 eq.) After 40 h of stirring at 80 °C, the mixture was precipitated from acetone, purified by Sephadex C-50 column, and the product was further recrystallized from MeOH. The yield was 12%. De los Reyes-Berbel et al. [199], mentioned at secondary amino γ-CDs, performed Michael addition of prepared ethanolamine-derived γ-CDs with divinyl sulfone (2 eq.) in water at rt. The product was precipitated from acetone after 1 h of stirring. After drying, the yield was 70%.

**Scheme 10:**
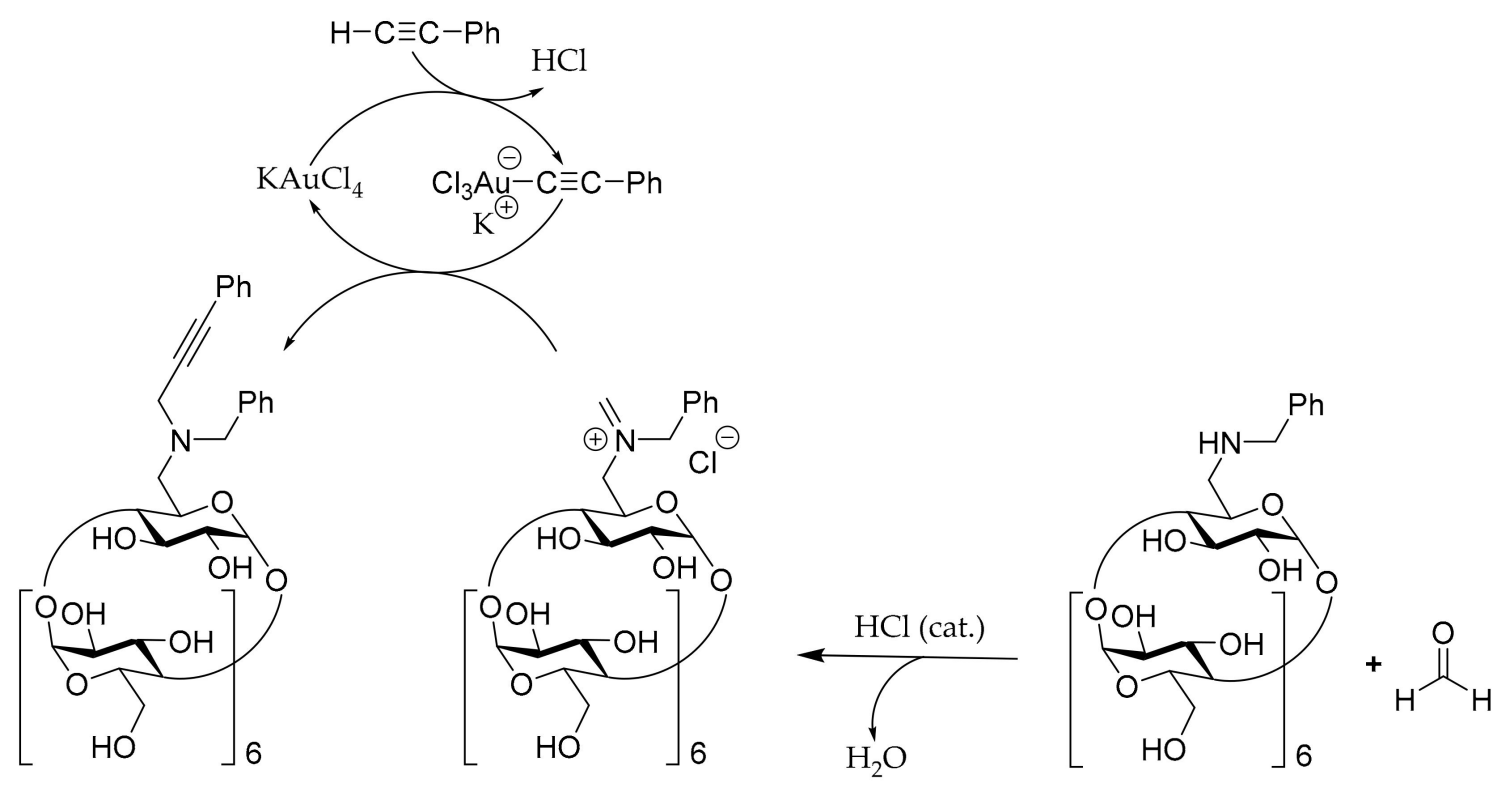
Proposed mechanism of Au(III)-catalyzed three-component coupling reaction (redrawn from Hui et al. [204]).

The last paragraph is devoted to notable examples which do not fit into previously describe categories. Samal and Geckeler [140] prepared unusual CD-fullerene derivatives. N_3_-α-CD or N_3_-β-CD was dissolved in DMSO, and fullerene C_60_ (1 eq.) in toluene was added in 6 h at 90 °C. One of the double bonds in the fullerene structure reacted with the azido group, and molecular nitrogen was released. The product was purified by membrane filtration and isolated in an 87% or 67% yield. Hui et al. [204] developed propargylamine modified β-CD synthesis by a gold(III) catalyzed three-component coupling reaction. Benzylamino-derived β-CD was mixed with formaldehyde (10 eq.) and KAuCl_4_ (2.5 mol%) in water and heated to 40 °C for 24 h. The resulting suspension was filtered, dissolved in boiling water, and reprecipitated after filtration of insoluble impurities. Yields were ranging from 26 to 67%. The proposed mechanism by the authors is depicted in Scheme 10.

#### 1.9.4. Quaternary Amines

Most authors utilized the reaction of Ts-CD or I-CD with a tertiary amine in DMF. No other solvent is used in published protocols. Binkowski et al. [205] synthesized a series of alkylammonium β-CDs. Moreover, they studied their self-inclusion behavior. I-β-CD was dissolved in DMF and 20 eq. of *N*-alkyl-*N*,*N*-dimethylamine was added. The solution was stirred for 24 h at 80 °C. The mixture was evaporated and suspended in an acetone/chloroform mixture. Insoluble product was separated by filtration and purified by ion-exchange column chromatography. The average yield of products was 40%. Tang et al. [184] performed the reaction of Ts-α-CD with 1-butylimidazole in DMF to substitute the tosyl group. After 2 days of stirring at 90 °C, the reaction mixture was evaporated, dissolved in MeOH/water mixture, and precipitated from ethyl acetate. The charged product was obtained in an 80% yield.

Wang et al. [206] have quarternized 6^A^-(6-aminohexyl)amino-6^A^-deoxy-γ-CD. The reaction was done in DMF with a large excess of MeI (200 eq.) After 15 h of stirring at 60 °C, the product was precipitated from acetone and isolated in a 75% yield. They tested the ability of the product to affect the stereochemical outcome in the supramolecular photodimerization of 2-anthracenecarboxylic acid.

Some authors prefer to do the reaction without any solvent if the appropriate amine is liquid. Wang et al. [207] prepared a methyl-substituted pyridinium β-CD derivative and further modified it to a cyanine-based dye and studied its spectral properties. Ts-β-CD was dissolved in 4-methylpyridine; the solution was heated to 85 °C and stirred for 12 h. The product was isolated by precipitation from acetone and isolated in a 90% yield. In the already mentioned article related to amino-CDs preparation [56], we used two different strategies to prepare quaternary amine CDs. In the first one, CD tertiary amines were further methylated by the reaction with MeI in DMF. Mixtures were stirred for 18 h at 30 °C and isolated by precipitation from acetone. Yields were between 80 and 90%. The second strategy concerned the reaction of Ts-CD with trimethylamine water solution (45 wt.%) in a sealed vessel. After stirring for 12 h at 80 °C, the product had to be purified by ion-exchange chromatography to separate it from the native CD and 3,6-anhydro-CD. The yields were 50–70%.

To summarize this chapter. Concerning H_2_N-CD, the most utilized method is Staudinger reduction of the corresponding azide by PPh_3_. It is necessary to have a proton source for the reaction. Due to that, the reaction is done in non-dry DMF, or DMF/water, or DMF/aqueous NH_3_ mixtures. A common disadvantage may be a trace amount of PPh_3_ complexed in the CD cavity.

Secondary and tertiary amines are most often synthesized by the reaction of Ts-CD with a primary amine in a solvent. The most utilized solvent is DMF and distantly followed by NMP and water. If the DMF is used, chemists should be aware of common dimethylamine impurity. This amine results from DMF decomposition caused by heating, and this amine could also react with Ts-CD, thus forming an undesired by-product. If possible, the amine being a liquid, the best option is to perform this reaction under solvent-free conditions. Some authors prefer to synthesize these CD derivatives by a reductive elimination strategy, utilizing 6^A^-deoxy-6^A^-oxo-CD and amine combination or H_2_N-CD and aldehyde. Reactions are primarily done in water solutions with NaBH_3_CN as a reducing agent.

Quaternary amines are usually synthesized by secondary or tertiary amino CD reactions with alkyl halides (mostly MeI).

Concerning purification, many authors purify their products by precipitation from acetone or EtOH. In many cases, this purification is entirely sufficient. However, if typical by-products as 3,6-anhydro-CD or native CD are formed, ion-exchange column chromatography is the best way to purify the product. By-products could be eluted by water or water/MeOH mixtures and amino products by aqueous NH_3_ or NH_4_HCO_3_ solutions. Excess of NH_4_HCO_3_ could be later decomposed to NH_3_ and CO_2_ by repeated evaporation from water.

Table 8 shows the number of articles using the various strategies described herein, along with yields and purification methods.

### 1.10. 6^A^-Deoxy-6^A^-oxo-CD

In the last chapter about alkylamino-CDs, a reductive amination reaction between 6^A^-deoxy-6^A^-oxo-CD and amine was mentioned. Therefore, methods for the preparation of 6^A^-deoxy-6^A^-oxo-CD should also be mentioned. No literature about 6^A^-deoxy-6^A^-oxo-γ-CD was found.

Concerning the 6^A^-deoxy-6^A^-oxo-α-CD, Huh et al. [208] synthesized this compound during their work on α-CD-conjugated poly(e-lysine)s and their inclusion complexation behavior studies. Native α-CD was dissolved in DMSO, 2 eq. of DMP were added, and the solution was stirred for 1 h at rt. The product was isolated by repeated precipitation from acetone. The yield was 88%. Due to the excess of the oxidizing agent and precipitation purification technique, the purity of the product is questionable.

Martin and Czarnik [209] published an article describing the preparation of 6^A^-deoxy-6^A^-oxo-β-CD in 1994. Ts-β-CD was dissolved in DMSO, and 10 eq. of collidine was added. Then, the solution was heated to 135 °C for 1.5 h. The aldehyde functional group was formed by the Nace reaction [210]. The product was isolated in 64% yield by precipitation from acetone and reprecipitation from EtOH. Bertolla et al. [211] utilized the same reaction during their synthesis of oxytocin-modified β-CD, a potential targeted drug carrier system. However, the authors performed the reaction in a microwave reactor. The mixture was irradiated for 20 min at 200 °C, and the product was purified by preparative LC/MS. The aldehyde was isolated in a 60% yield.

Swamy et al. [212] used the IBX oxidation strategy to work on CD–dipyrromethane conjugates synthetic methodology. The authors performed the oxidation of native β-CD in DMF. A slight excess of IBX (1.5 eq.) was added, and the solution was stirred for 24 h at rt. The product was precipitated from acetone and isolated in a 95% yield. The authors acknowledged unreacted β-CD impurity in their product and purified the mixture after the next reaction step.

### 1.11. 6^A^-[(Alkyl)imino]-6^A^-deoxy-β-CD and 6^A^-[(Alkylmethylene)amino]-6^A^-deoxy-CD

When we mentioned reductive amination reactions and 6^A^-deoxy-6^A^-oxo-CD preparations, the chapter concerning imino-CD derivatives should follow. There are two types of these derivatives, which differ by the position of the imine bond because chemists can use H_2_N-β-CD/aldehyde or 6^A^-deoxy-6^A^-oxo-β-CD/amine combination. Interestingly, a lesser number of derivatives are prepared from H_2_N-β-CD.

Deunf et al. [213] synthesized and investigated an electrochemical behavior of cobalt(II)-CD complex. For the synthesis of the CD ligand, the reaction between H_2_N-β-CD and 2-pyridine-carboxaldehyde (1 eq.) was performed. Starting compounds were dissolved in absolute EtOH, and the solution was refluxed for 2 h. The precipitate was formed during the reaction. After filtration and drying, the product was isolated in an 80% yield.

Liu et al. [214] developed calix[4]arene-β-CD monomer and dimer and studied their ability to form supramolecular sandwich complexes. Both compounds were prepared by the reaction of 6^A^-deoxy-6^A^-oxo-β-CD with amino-modified calix[4]arenes in DMF or DMF/MeCN mixture. Reactions were stirred at elevated temperatures (75–90 °C) for several days (2–7). Products were purified by column chromatography and isolated in 10% yields.

Malenkovskaya et al. [215] published a protocol for 6^A^-deoxy-6^A^-oxo-β-CD preparation and its utilization in imine formation reactions. Imino-β-CD derivatives were synthesized using the following protocol. 6^A^-Deoxy-6^A^-oxo-β-CD was dissolved in DMSO/benzene 1/2 mixture, and the solution was refluxed with a Dean-Stark trap until the separation of water ceased. Benzene was distilled off, and appropriate amine (1 eq.) was added. The solution was heated to 80 °C and stirred for 10 h. Products were isolated by precipitation from acetone with a yield of around 50%.

Yoon et al. [196] published a synthetic protocol for the 6^A^-deoxy-6^A^-oxo-β-CD synthesis and subsequent carboxylic acid derivative. Oxime and hydrazone formation from 6^A^-deoxy-6^A^-oxo-β-CD were tested. Reactions were done in 50% aqueous NH_2_OH or water with 1.1 eq. of hydrazine hydrate. Solutions were stirred for a couple of hours at rt. Then, the products were precipitated from EtOH or iPrOH and isolated in yields 43 and 60%.

To summarize this chapter, these are all the equilibrium reactions. Therefore, the equilibrium needs to be pushed to support product formation. According to our experience, the best option is to use a significant excess of the non-CD starting compound (aldehyde or amine, depending on the CD). Otherwise, it will probably not be possible to push the reaction to completion, and a starting CD derivative will still contaminate the isolated product. Readers can compare the vast differences between yields of products isolated by simple precipitation and column chromatography from the cited articles.

### 1.12. 6^A^-N-Acyls of CDs

**Scheme 11:**
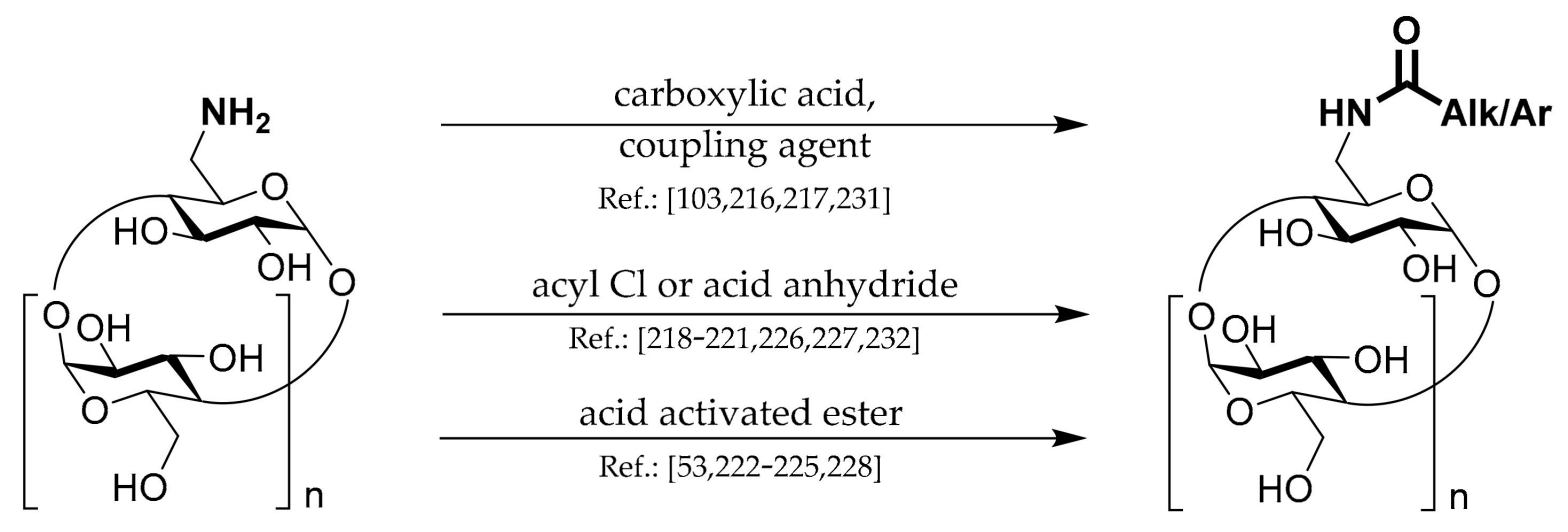
General methods for the preparation of 6^A^-*N*-acyls of CDs.

The number of articles describing this type of derivatives is too large, so only selected papers with original synthetic procedures, or in some sense unconventional, are presented here. The most common and general methods are depicted in Scheme 11.

Four main synthetic strategies could be found for the synthesis of 6^A^-(acylamino)-6^A^-deoxy-CDs. Most chemists utilize a coupling agent strategy to connect H_2_N-CD with carboxylic acid derivatives. The most often used coupling agents are DCC/HOBt combination, BOP, PyBOP, DMTMM, HATU, HBTU, EDC/HOBt, and DIC/HOBt. In basically all cases, reactions are done in DMF. The stoichiometric amount is usually between 1–2 eq. (calc. for CD). Miyauchi and Harada [216] prepared supramolecular polymers having α- and β-CD units. These CD units were synthesized by acylation reactions. The reaction between Boc-protected cinnamoyl acid (2 eq.) and H_2_N-α-CD was done in DMF. DCC and HOBt (1.3 eq. for both) were added, and the mixture was stirred for 5 days at rt. The product was isolated in a 49% after size exclusion gel chromatography. Leray et al. [217] performed the first enzymatic coupling of galactose to 2-acetamido-2-deoxy-glucose-β-CD derivative prepared by the reaction of H_2_N-β-CD with 2-acetamido-2-deoxy-β-D-glucopyranosyl-hexanoic acid (1 eq.) in DMF. DCC/HOBt reagents (1.3 eq.) were added, and the mixture was stirred at rt. The product was purified by classical silica gel column chromatography and isolated in a 28% yield. Ueno et al. [103] synthesized several pyrene-appended γ-CD and studied their self-inclusion behavior and dimers formation in their original paper from 1989. One of these compounds was synthesized by the reaction of 4-(1-pyrene)butyric acid (4.5 eq.) with H_2_N-γ-CD in DMF with DCC (5 eq.) After 5 h of stirring at rt, the mixture was precipitated from acetone and purified by Sephadex column chromatography in the DMF/water mixture. The yield of the product was 18%.

The second popular synthetic strategy is the reaction with acyl chloride. In several papers, the authors perform the reaction of acryloyl chloride with H_2_N-CD in an aqueous NaHCO_3_ solution to synthesize monomers for polymerization reactions. Harada et al. [218] prepared acrylamide-based gels functionalized with either CD host or small organic guest molecules and studied their self-assembly on a macroscopic level. The monomer was synthesized from acryloyl chloride in aqueous NaHCO_3_ at pH 10 (adjusted by NaOH). The mixture was stirred for 6 h at 0 °C. The product was precipitated from acetone and further purified by RPC. The yield was 79%. Meyer et al. [219] have synthesized amido β-CD derivatives possessing double or triple bonds. Then, they used their inclusion complexaction ability to control the regioselectivity of nitril oxide cycloadditions with the CD double or triple bond. The amido β-CD derivatives were synthesized by the reaction with propynoyl, propenoyl, or (*E*)-3-ethoxycarbonylpropenoyl chloride (1.1 eq.) in an aqueous NaOH (1.1 eq.) solution. After stirring for more than 12 h at rt, the mixture was precipitated from acetone and purified by ion-exchange column chromatography. Yields were between 70 and 80%.

Solvents other than water were also used for reactions with acyl chlorides. Surpateanu et al. [220] synthesized tetrathiafulvalene-β-CD derivative and studied its complexation with adamantanol. The reaction was done in DMF/pyridine mixture with 4,5-ethylene-dithio-4´-chlorocarbonyl-tetrathiafulvalene (1 eq.) The solution was stirred at 65 °C for 14 h. The mixture was precipitated from acetone and further purified by Sephadex column chromatography. The product was obtained in a 47% yield.

Maeda et al. [221] synthesized phenylacetylenes bearing α-, β-, or γ-CD and studied their helical polymers. The monomers were synthesized by the reaction of (4-carboxyphenyl)acetylene chloride in THF in the presence of Na_2_CO_3_. After 4.5 h of stirring at rt, the mixture was neutralized by HCl, precipitated, and products isolated in about 70% yield.

The third strategy is the reaction of activated esters with H_2_N-CD. Most authors synthesize *N*-hydroxysuccinimide and 4-nitrophenyl esters. Becuwe et al. [222] synthesized fluorescent indolizidine-β-CD derivative and tested its potential in sensor applications. Indolizidine 4-nitrophenyl ester derivative (1 eq.) was mixed with H_2_N-β-CD in DMF and stirred for 12 h at 60 °C. The mixture was precipitated by pouring into acetone, and the solid was purified by Sephadex column chromatography. The yield of the product was 22%. Takahashi et al. [223] synthesized an α-CD-β-CD dimer and cinnamide guest dimers and studied their ability to form a pinching-type or supramolecular polymer-type complex based on the guest type. The β-CD precursor was prepared by the reaction with terephthalic acid methyl *N*-hydroxysuccinimide ester (1.1 eq.) in DMF. After stirring at rt for 36 h, the product was isolated and purified by precipitation from acetone. The yield was 87%.

Only two articles describing the synthesis with activated esters could be found for γ-CD. Pham et al. [53] published a methodology for preparation γ-CD including succinimide linked. H_2_N-γ-CD reacted with prepared bis-4-nitrophenyl succinate (0.4 eq.) in pyridine for 48 h at rt. The mixture was precipitated from diethyl ether. The product was further purified by ion-exchange column chromatography and isolated in a 91% yield. Tsumoto et al. [224] synthesized a fullerene C_60_-γ-CD conjugate to make the fullerene water-soluble. Then they tested the conjugate’s biological activity. Fullerene *N*-hydroxysuccinimide derivative (0.7 eq.) was mixed with H_2_N-γ-CD in DMF and stirred for 48 h at rt. The product was purified by precipitation from MeOH and isolated in a 78% yield.

Some authors have used uncommonly encountered activated esters. Yan et al. [225] have prepared supramolecular dendrimers and studied their thermoresponsive behavior. β-CD trimer served as the dendrimer core, and adamantane/oligo ethylene glycol-based threefold dendritic guests served as dendrons. The β-CD trimer was prepared by the reaction of 1,3,5-benzenetricarbonyl tris(pentafluorophenyl)ester (6 eq.) with H_2_N-β-CD in DMF with DIPEA (7 eq.) The mixture was stirred at rt for 24 h. The product was purified by precipitation from acetone and subsequent dialysis against water for 2 days and isolated in a 15% yield.

Utilization of anhydrides is another strategy. Onagi et al. [226] synthesized several α-CD amides derived from stilbene and studied its rotation in the CD cavity due to the formation of intramolecular inclusion complexes. The starting CD amido derivative was prepared by the reaction with succinic anhydride (1 eq.) in DMF. The reaction was stirred for 100 h at rt. After column chromatography purification, the product was isolated in an 86% yield.

Only a small number of authors use anhydrides for the synthesis of amido-β-CD derivatives. Yan et al. [227] synthesized an EDTA-β-CD dimer and utilized its Ce(IV) complex as a catalyst for hydrolysis of bis(4-nitrophenyl) phosphate. The dimer was prepared by the reaction of 4,4’-(ethane-1,2-diyl)bis[morpholine-2,6-dione (EDTA dianhydride) with H_2_N-β-CD in DMF. After 24 h of stirring at rt, water was added, and the mixture was heated for 5 h at 80 °C. The dimer was later purified by RPC and isolated in an 85% yield.

Some authors prepare first an activated ester derived from a desired carboxylic acid and then perform the reaction with H_2_N-CD. Wyness et al. [228] synthesized coronand-linked α- and β-CD and studied their inclusion complexes with anionic organic molecules. The authors prepared and isolated 4-nitrophenyl esters of bis(carboxymethyl)azacrown ether derivative. Then it was added into H_2_N-α-CD DMF solution, and the mixture was stirred for 18 h at rt. The product was purified by ion-exchange column chromatography and isolated in a 16% yield.

Some uncommon protocols should also be mentioned. Zhao et al. [229] studied the inclusion complexation behavior of morin hydrate and quercetin with two amide-bridge β-CD dimers. These dimers were synthesized by the reaction of Ts-β-CD with 1,3-malonamide or 1,4-succinamide in DMF. The solution was stirred for 48 h at 90 °C. The mixture was precipitated from acetone and further purified by Sephadex column chromatography. The yield of the products was 17 and 10%. Mallard et al. [230] synthesized a series of anthracene appended β-CD and studied the influence of the amino, amido, and triazole linker on the inclusion abilities. The amido-derivative was prepared by the reaction with anthracene-9-carbonyl fluoride (prepared by the reaction of the acid with cyanuric fluoride) in DMF. The mixture was stirred for more than 12 h at rt, precipitated from acetone, and purified by Sephadex column chromatography. The product was obtained in a 73% yield.

In contrast to α- and γ-CD derivatives, in the case of β-CD, one can also find literature describing disubstituted amides synthesis. Some authors utilize the coupling agent strategy. Malanga et al. [231] developed a new synthetic strategy for xanthene-appended β-CD. It is based on the rhodamine B reaction with H_2_N-β-CD and in situ formation of spirolactam moiety. The reaction was done in water with 1 eq. of rhodamine B, 4 eq. of *N*-methyl morpholine, and 1 eq. of DMTMM. The solution was stirred for 3 h at rt. After precipitation from acetone, the crude was purified by column chromatography and isolated in a 72% yield.

Other authors utilize the reaction of the secondary amino-β-CD derivative with acyl chloride, as Coulston et al. [232] who did to synthesize molecular machines prototypes. Based on phenylpropionamido and cinnamido-β-CDs, these machines can repeatedly form complexes with adamantanol. This molecular recognition energy is harnessed to do work and constrain the geometry of an amide bond. Photoisomerization of the cinnamide double-bound could work as an on/off switch. The synthetic procedure is based on the reaction of 6^A^-deoxy-6^A^-methylamino-β-CD with hydrocinnamoyl or trans-cinnamoyl chloride (1.1 eq.) in DMF with TEA (4 eq.) The mixture was stirred for 6 h at rt. Products were then purified by precipitation from acetone and subsequent anion- and cation-exchange columns. Yields were 60 and 12%.

To summarize the chapter. From the published data, it is clear that most chemists prepare 6^A^-(acylamino)-6^A^-deoxy-CDs by the reaction of H_2_N-CD with a carboxylic acid in the presence of suitable coupling reagents, which were mentioned thorough the chapter. DCC/HOBt combination is the most used one. With only a few exceptions, all described procedures are done in DMF. The synthesis of activated ester and its reaction with H_2_N-CD forms the second largest group of published articles on this topic. 4-Nitrophenyl and *N*-hydroxysuccinimide esters are utilized in the majority of cases. However, even pentafluorophenyl esters could be found.

Acyl chlorides are also popular reagents for CD amides synthesis—particularly acryloyl chloride, which is used very often to synthesize CD monomers that are further polymerized. Anhydrides are not so commonly used. The only exceptions are succinic and acetic anhydrides. Synthesis of CD amides from common amides as 1,3-malonamide by nucleophilic displacement of the leaving group situated on CD are rare but could be found. For β-CD, even disubstituted amides synthesis is described. Concerning the purification, precipitation is often utilized as the only technique and should be sufficient for many cases, especially for coupling agent by-products removal. However, if the excess of the H_2_N-CD was used, the residues could be purified by ion-exchange column chromatography. A possible side-reaction could be the esterification reaction with some CD hydroxyl groups. However, in most cases, these are hydrolyzed much more readily compared to amido moieties. Therefore, adding an excess of NaOH will hydrolyze ester groups, and amido-CD can be purified much easier.

Table 9 describes a number of articles using the various strategies described herein, along with yields and purification methods.

### 1.13. 6^A^-N-Thioureas of CDs

Only four articles with thoroughly described synthesis could be found. However, only the two oldest and original papers will be mentioned.

Benito et al. [233] prepared dendron-like β-CD by the reaction of H_2_N-β-CD with different isothiocyanate-armed α-d-mannopyranosyl dendrons (1 eq.) in pyridine or aqueous NaHCO_3_ solution. Reactions were stirred for 12 to 24 h at a temperature ranging from 25 to 60 °C. Products were purified by column chromatography and isolated in yields from 40 to 70%. Synthesized compounds were tested as potential drug delivery compounds utilizing a sugar-directed strategy.

Aime et al. [234] synthesized thioureido-tethered β-CD dimer by aza-Wittig reaction utilizing polymer-bound PPh_3_ and microwave irradiation. The dimer was adducted with Gd(III) chelates, and its stability constants and relaxivity values were determined, testing the potential for MRI applications. The synthesis was done as follows: N_3_-β-CD was dissolved in DMF and mixed with polymer-bound PPh_3_, CS_2_ (5 eq.) was added in DMF. The mixture was placed into a microwave reactor and irradiated for 30 min at 110 °C. After filtration, DMF was evaporated, and the solid was washed with diethyl ether. The yield of the product was 93%.

Examples of the use of β-CDs bound by thiourea linkers can be found in the recent review about CD based functional glyconomaterials [235].

No thiourea α- and γ-CD derivatives synthesis were described.

### 1.14. 6^A^-N-Ureas of CDs

Cieslinski et al. [236] synthesized α- and β-CD homo- and heterodimers linked via urea linker. Then they studied their complexation with anionic organic molecules. The general procedure was a dissolution of N_3_-CD in DMF saturated with CO_2_, 1.7 eq. of PPh_3_ was added. The mixture was stirred for 27 h at rt while CO_2_ was continuously bubbled through the solution. The mixture was poured into acetone, and the solid crude was purified by Sephadex column chromatography. Yields were ranging between 50 and 70%.

Pham et al. [53] applied the Cieslinski method for the preparation of γ-CD analog dimer. The product was obtained in a 53% after precipitation from acetone.

Sallas et al. [197] published the synthesis of the urea-β-CD dimers already in 1998 and studied their metal complexation abilities. The synthesis was the same as described by Cieslinski. The authors used the aza-Wittig reaction again, and after repeated precipitation from acetone, the product was obtained in a 96% yield.

Charbonnier et al. [237] developed a new methodology based on peracetylated N_3_-β-CD and its transformation to isocyanate. The compound could be synthesized by the reaction of peracetylated N_3_-β-CD in toluene saturated with CO_2_. Then, 1.4 eq. of PPh_3_ was added, and the mixture was stirred for 20 h at rt while it was continuously bubbled with CO_2_. The product was obtained in 92% yield after precipitation from cyclohexane. This β-CD derivative can further react with different amines to form urea-modified β-CDs. The authors tested piperazine and aza-crown derivatives. Reactions were done in toluene at rt for 24 h, and products could be isolated in moderate (51%) to high (94%) yields after precipitation from cyclohexane.

### 1.15. 6^A^-N-Carbamates of CDs

No articles describing γ-CD carbamate synthesis could be found.

Hanessian et al. [151], in their paper dealing with selective modification of CDs, described the protection of H_2_N-α-CD by benzyloxycarbonyl protection group. The reaction was done in an aqueous NaHCO_3_/DCM mixture, 10 eq. of benzyl chloroformate was added. The biphasic mixture was stirred for 12 h at rt, the organic phase was separated, and the aqueous one was purified by RPC. The yield of the CBz-protected H_2_N-α-CD was 87%.

Onagi et al. [226], mentioned in the chapter about amido-CDs derivatives, installed Boc protecting group into H_2_N-α-CD. Di-*tert*-butyl dicarbonate (5 eq.) was added into H_2_N-α-CD DMF solution together with TEA (1.1 eq.) The solution was stirred for 24 h at rt, evaporated, washed with water, and freeze-dried. The protected amino-α-CD was obtained in a 53% yield.

Ghera et al. [238] published the article about utilizing olefin metathesis reaction for further and efficient CD modification. As a starting compound for these reactions, Boc-protected 6^A^-allylamino-6^A^-deoxy-β-CD was prepared. The reaction was done in MeOH; di-*tert*-butyl dicarbonate (1.2 eq.) and NaHCO_3_ (3 eq.) were added. The solution was ultra-sounded for 12 h, and the product was purified by reverse-phase column chromatography and isolated in a 67% yield.

Not only chlorides or dicarbonates could be used to form the carbamate group in CD derivatives. White et al. [239] published a paper dealing with β-CD biofunctionalization and its peptide modification. During the desired compounds synthesis, H_2_N-β-CD had to be protected by Cbz protecting group. The reaction was done in water with NaHCO_3_ (1 eq.); *N*-(benzyloxycarbonyloxy)succinimide (1 eq.) in dioxane was added, and the mixture was stirred for 4 h at rt. The mixture was evaporated, and the product was recrystallized from water. The yield of the product was 51%.

### 1.16. 6^A^-O-Carbonates of CDs

Only one article describing the synthesis of mono-substituted carbonate β-CD could be found. No articles concerning α- and γ-CD carbonate synthesis could be traced.

Huang et al. [240] published a paper about the novel synthesis of ethyl carbonate β-CD. Dried β-CD was dissolved in dry DMF; K_2_CO_3_ (1.25 eq.) was added, and the solution was warmed up to 110 °C. Then, 14 eq. of diethyl carbonate was added for 15 min. The mixture was evaporated and purified by a semi-preparative reverse-phase column. The product was isolated in 66% yield together with 11% of disubstituted and 9% of trisubstituted ethyl carbonate β-CD.

### 1.17. 6^A^-C-Aryls/alkyls of CDs

This part could be considered a curiosity because there is only one article with a fully described synthesis. Swamy et al. [212] published a methodology for CD-dipyrromethane derivative by the condensation reaction of an aldehyde with pyrrole. The reaction was done in pyridine, and 42 eq. of pyrrole was added to the 6^A^-deoxy-6^A^-oxo-β-CD. The solution was stirred for 2 h at 70 °C; then, TFA (0.6 eq.) was added, and the mixture was further stirred for 24 h. The final product was isolated by reverse-phase column chromatography and obtained in a 41% yield.

### 1.18. 6^A^-Se-Aryls/alkyls and 6^A^-Te-Aryls/alkyls of CDs

This chapter can be considered unique, as these derivatives are uncommon in CD chemistry. Only two articles could be found.

Liu et al. [241] prepared a series of β-CD dimers connected by organoselenium linkers and studied their inclusion complexation behavior with fluorescent dyes. The dimer connected via primary CD rims was prepared by the reaction of 1,2-diseleno cyclopentane with Ts-β-CD. 1,2-Diseleno cyclopentane (0.5 eq.) was dissolved in absolute EtOH, NaOH (1.5 eq.), and NaBH_4_ (1.5 eq.) were added. When the solution became colorless, Ts-β-CD in DMF was added to the generated sodium cyclopentane selenolate, and the mixture was stirred at 80 °C for 5 h. The product was purified by Sephadex column chromatography and isolated in a 53% yield.

McNaughton et al. [37] synthesized β-CD tellurides and evaluated their capacity to catalyze the reduction of hydrogen peroxide organic hydroperoxides in the presence of glutathione, NADPH, and GSSG-reductase to study their potential as glutathione peroxidase mimics. The synthetic strategy was similar to Liu’s. Sodium organyltellurolate (prepared from ditelluro dialkyl/diaryl and NaBH_4_) in absolute EtOH was combined with Ts-β-CD water solution. The mixture was stirred for 16 h at 60 °C. The solution was extracted between DCM and water. The formed precipitate in the aqueous phase was filtered and washed. Yields were ranging from 23 to 62%. The authors even prepared a Te-β-CD dimer by the reaction of sodium telluride (0.5 eq.) with Ts-β-CD in water. After stirring for 16 h at 60 °C, the product was precipitated from EtOH and recrystallized from water/EtOH mixture. The dimer was isolated in a 54% yield.

## 2. Conclusions

From the point of view of organic synthetic chemists, we attempted to give an overview of the most valuable methods for preparing mono-6-substituted CD derivatives and warn of potential problems. We separated the methods into sections by the type of the derivatives and sorted them by the synthetic logic—intermediates first. To conclude the review, we summarize the most critical findings that are noteworthy.

Of course, the purity of the final products depends on the purity of the intermediates. By a vast majority, the most used intermediates are Ts-CD derivatives, for which many methods have been developed. Nevertheless, by most of them, up to 30% yield can be achieved, and the most crucial part of the preparation is the proper purification, which is often neglected. After direct monoderivatization of any CD, the reaction mixture will contain unreacted CD, the monosubstituted CD, and di or more substituted CD side-products. For Ts-β-CD, the easiest and most efficient method for separation is repeated crystallization from water/alcohol mixtures. For α- and γ-CDs, the preparative LC on a reversed-phase column is the most helpful.

The reaction of Ts-CD with various nucleophiles, such as amines, thiolates, aryloxides, carboxylates, or azide, can give the product in a high yield. The most common by-products can be prevented by using either solvent-free conditions or dried solvents. Attention should be paid to DMF, which is often used as a solvent with tosyl derivatives of CD but may decompose to dimethylamine at higher temperatures. This strong nucleophile then provides the corresponding dimethylamine by-products. 

Another common side-product, usually unexpected by chemists inexperienced in CD chemistry, is the CD analog containing one 3,6-anhydroglucose unit. It is formed by intramolecular cyclization of the glucose unit, which had the tosyl group. It takes place in the presence of a strong base, which is capable of deprotonating the 3-OH group. Another consequence of this behavior is the practical inability to prepare 6^A^-*O*-alkyl CD derivatives by reacting the alkoxide with Ts-CD.

The 6^A^-*O*-Alkyl-CDs are prepared either by direct alkylation of native CD or by a series of selective protection/deprotection steps. The most used series of reactions used for this purpose consists of perbenzylation, selective 6^A^-*O*-debenzylation, alkylation, and full debenzylation. Despite looking quite laborious, this strategy offers high yields and easy separation in each step. It might be the method of choice when the separation of the reaction mixture after direct alkylation is difficult.

Another heavily used intermediate for the preparation of 6^A^-substituted CD derivatives is the N_3_-CD. It can be easily prepared from Ts-CD in high yields by reacting with even one or two equivalents of NaN_3_ in water. It is then used for CuAAC click reactions with alkynes, usually catalyzed by the CuSO_4_/NaASC mixture, giving the products in satisfactory yields. The NH_2_-CD is another intermediate made generally by reducing the N_3_-CD, used to prepare CD amine derivatives such as amides, carbamates, or carbonates.

We hope this review will help those looking for easy covalent incorporation of cyclodextrins into more complicated systems or those looking for an overview of applications in which the described CD derivatives could be utilized.

## Figures and Tables

**Table 1:** Preparation of 6^A^-*O*-aryl/alkyl-sulfonates of CDs.

Synthetic Method	Number of Articles	Yield
heterogenous method	6	22% ^a^ [36], 32% ^b^ [37], 35% ^b^ [38], 54% ^b^ [52], 58% ^b^ [39], 1% ^c^ [30]
homogeneous method	4	6% ^a^ [34], 13% ^a^ [23], 34% ^a^ [35], 11% ^b^ [33]
pyridine method	13	26% ^a^ [40], 40% ^a^ [26], 69% ^b^ [54], 15% ^c^ [28], 47% ^c^ [24], 3.4% ^d^ [41]
TsIm	2	40% ^b^ [43], 55% ^b^ [44]
Ts_2_O	1	61% ^b^ [45]
heterogenous method and Cu(II) salt	1	35% ^a^ [48]
CD tosylation with Bu_2_SnO in DMF	1	5% ^c^ [53] (2^A^-*O*- regioisomer also prepared)
more doses of TsCl	2	25% ^a^ [46], 31% ^a^ [47]

Purification by ^a^ recrystallization, ^b^ precipitation, ^c^ column chromatography, ^d^ preparative TLC.

**Table 2:** Preparation of 6^A^-deoxy-6^A^-halogens of CDs.

Synthetic Method	Number of Articles	Yield
Ts-CDylation with organic or inorganic halide salt	6	85% ^a^ [59], 90% ^b^ [61], 89% ^c^ [24]
Appel reaction with CD	1	31% ^c^ [58]
(de)benzylation	1	70% ^c^ [57]

Purification by ^a^ recrystallization, ^b^ precipitation, ^c^ column chromatography.

**Table 3:** Preparation of 6^A^-*O*-aryls/alkyls of CDs.

Synthetic Method	Number of Articles	Yield
(de)benzylation	3	77% ^a^ [64], 84% ^a^ [63], 51% ^d^ [65]
direct alkylation	9	13% ^a^ [75], 34% ^a^ [69], 6% ^b^ [72], 13% ^b^ [66], 50% ^c^ [68]
Ts-CDylation	8	10% ^a^ [82], 61% ^a^ [78], 78% ^c^ [77]
photolysis	1	10% ^a^ [84]

Purification by ^a^ column chromatography; ^b^ peracetylation, column chromatography, and deacetylation; ^c^ precipitation, ^d^ preparative TLC.

**Table 4:** Preparation of 6^A^-*O*-acyls of CDs.

Synthetic Method	Number of Articles	Yield
Ts-CDylation of carboxylate	12	5% ^a^ [105], 76% ^a^ [109], 59% ^b^ [112], 24% ^c^ [114], 42% ^c^ [108]
native CD with acyl chloride and its analogs	14	5% ^a^ [92], 15% ^b^ [94], 86% ^b^ [100], 7% ^c^ [103], 18% ^c^ [98]
native CD with carboxylic acid and coupling agent	7	13% ^a^ [91], 28% ^a^ [88], 15% ^b^ [89], 70% ^b^ [85]
(de)benzylation	1	82% ^a^ [116]
native CD with acyl chloride in the presence of Cu(II) salt	1	31% ^a^ [119]
acid activated ester	1	80% ^c^ [118]

Purification by ^a^ column chromatography, ^b^ precipitation, ^c^ crystallization.

**Table 5:** Preparation of 6^A^-*S*-aryls/alkyls/acyls of CDs.

Synthetic Method	Number of Articles	Yield
Ts-CDylation of thiolate	13	20% ^a^ [126], 88% ^a^ [129], 31% ^b^ [122], 94% ^b^ [121], 66% ^c^ [120], 98% ^d^ [128], 85% ^e^ [127]
X-CD with thiolate	6	20% ^a^ [131], 36% ^a^ [130], 46% ^a^ [132], 49% ^a^ [134], 67% ^a^ [61], 80% ^a^ [58]
HS-CD with alkyl halide or Michael acceptor	2	48% ^a^ [136], 58% ^c^ [135]
CD with thiolate under Mitsunobu reaction conditions	1	28% ^a^ [138]
Ts-CDylation of thiolate under ball milling technique	1	73% ^a^ [139]

Purification by ^a^ column chromatography, ^b^ precipitation, ^c^ crystallization, ^d^ dialysis, ^e^ ultrafiltration.

**Table 6:** Preparation of 6^A^-deoxy-6^A^-azides of CDs.

Synthetic Method	Number of Articles	Yield
Ts-CDylation of NaN_3_ in water	11	81% ^a^ [147], 29% ^b^ [141], 90% ^b^ [140], 58% ^c^ [143], 80% ^d^ [145], 85% ^e^ [26]
Ts-CDylation of NaN_3_ in DMF	3	93% ^a^ [27], 99% ^c^ [35], 84% ^d^ [148]
Ts-CDylation of NaN_3_ with KI in DMF	1	88% ^b^ [33]
Ts-CDylation of NaN_3_ in DMF/water mixture	2	90% ^a^ [150], 93% ^c^ [149]
Ts-CDylation of NaN_3_ in DMF under microwave irradiation	1	75% ^c^ [44]
Native cyclodextrin with CBr_4_/PPh_3_/LiN_3_ (Appel reaction)	1	22% ^a^ [151]
Ts-CDylation of NaN_3_ under ball milling technique	1	90% ^c^ [139]
Ts-CDylation of LiN_3_ in water	1	89% ^c^ [152]
Ts-CDylation of NaN_3_ in EtOH	1	92% ^b^ [153]
X-CD with NaN_3_ in DMF under microwave irradiation	1	80% ^a^ [57]
Trips-CD with NaN_3_ in DMF	1	94% ^b^ [54]

Purification by ^a^ column chromatography, ^b^ precipitation, ^c^ crystallization, ^d^ dialysis, ^e^ tetrachloroethane complex formation.

**Table 7:** Preparation of 1,4-substituted triazoles of CDs.

Synthetic Method	Number of Articles	Yield
CuSO_4_·5H_2_O and sodium ascorbate	14	10% ^a^ [161], 80% ^a^ [57], 87% ^b^ [156], 70% ^c^ [158], 80% ^c^ [163], 95% ^d^ [164]
CuSO_4_·5H_2_O and sodium ascorbate under microwave irradiation	2	40% ^a^ [166], 60% ^b^ [165]
CuI	4	28% ^a^ [172], 70% ^a^ [171], 35% ^b^ [155], 64% ^b^ [170]
CuI under microwave irradiation	1	60% ^a^ [174]
CuBr	2	67% ^b^ [168], 92% ^c^ [167]
CuBr under microwave irradiation	1	30% ^a^ [169]
CuCl	1	30% ^a^ [173]
Cu(I) P-complexes	3	33% ^a^ [174], 72% ^b^ [175], 70% ^c^ [78]
metal copper	2	70% ^a^ [177], 81% ^a^ [178]
(MeCN)_4_Cu(I)PF_6_	2	90% ^a^ [142], 92% ^d^ [176]
Cu(I) solid support catalyst	2	95% ^b^ [179], 99% [180]

Purification by ^a^ column chromatography, ^b^ precipitation, ^c^ crystallization, ^d^ dialysis.

**Table 8:** Preparation of 6^A^-amines and aryl/alkylamines of CDs.

Synthetic Method	Number of Articles	Yield
**6^A^-Amino-6^A^-deoxy-cyclodextrin (H_2_N-CD)**		
Ts-CD with NH_3_	2	18% ^a^ [53], 70% ^b^ [25]
hydrogenation H_2_/Pd/C	2	87% ^d^ [33], 90% ^d^ [151]
hydrogenation NH_2_NH_2_/Pd/C	1	90% ^c^ [35]
Staudinger reduction PPh_3_	2	94% ^b^ [184], 98% ^b^ [144]
indium with NH_4_Cl	1	94% ^b^ [185]
cyclodextrin phthalimide reaction (Mitsunobu reaction)	1	41% ^a^ [183]
**Secondary 6^A^-aryl/alkylamines of CDs**		
Ts-CD with primary amine in a solvent	6	19% ^a^ [186], 21% ^a^ [190], 25% ^a^ [33], 39% ^a^ [198], 74% ^a^ [189], 88% ^a^ [199]
Ts-CD with primary amine under solvent-free conditions	4	70% ^a^ [56], 95% ^a^ [188], 37% ^b^ [200], 88% ^b^ [96]
I-CD with primary amine in a solvent	2	23% ^a^ [191], 80% ^a^ [192]
H_2_N-CD alkylation	3	13% ^a^ [193], 60% ^b^ [195], 75% ^b^ [194]
H_2_N-CD reductive amination	1	70% ^a^ [144]
6^A^-deoxy-6^A^-oxo-cyclodextrin reductive amination	1	88% ^a^ [196]
Mitsunobu reaction	1	2% ^a^ [197]
**Tertiary 6^A^-aryl/alkylamines of CDs**		
Ts-CD with secondary amine in a solvent	1	49% ^a^ [201]
Ts-CD with secondary amine under solvent-free conditions	3	75% ^a^ [36], 86% ^a^ [56], 95% ^a^ [188]
H_2_N-CD or secondary 6^A^-aryl/alkylamines of CDs alkylation	3	12% ^a^ [203], 25% ^a^ [202], 70% ^b^ [199]
Au(III)-catalyzed three-component coupling reaction	1	67% ^b^ [204]
N_3_-CD double bond reaction (aziridine formation)	1	87% ^e^ [140]
**Quaternary 6^A^-aryl/alkylamines of CDs**		
Ts-CD with tertiary amine under solvent-free conditions	2	58% ^a^ [56], 90% ^b^ [207]
Ts-CD or I-CD with secondary amine in a solvent	2	40% ^a^ [205], 80% ^b^ [184]
H_2_N-CD or tertiary 6^A^-aryl/alkylamines of CDs alkylation	2	75% ^b^ [206], 90% ^b^ [56]

Purification by ^a^ column chromatography, ^b^ precipitation, ^c^ crystallization, ^d^ filtration, ^e^ membrane filtration.

**Table 9:** Preparation of 6^A^-*N*-Acyls of CDs.

Synthetic Method	Number of Articles	Yield
coupling agent	4	18% ^a^ [103], 28% ^a^ [217], 49% ^a^ [216], 72% ^a^ [231]
acyl chloride	6	47% ^a^ [220], 60% ^a^ [232], 80% ^a^ [219], 76% ^b^ [221]
acid anhydride	2	85% ^a^ [227], 86% ^a^ [226]
acid activated ester	6	16% ^a^ [228], 91% ^a^ [53], 87% ^b^ [223], 15% ^c^ [225]
Ts-CD with amide	1	17% ^a^ [229]
acyl fluoride	1	73% ^a^ [230]

Purification by ^a^ column chromatography, ^b^ precipitation, ^c^ dialysis.

## Data Availability

The data presented in this review are openly available at https://cyclodextrins.org/synthesis-of-mono-6-substituted-cyclodextrins (accessed on 15 July 2021).
